# Mechanical Cell Reprogramming on Tissue-Mimicking Hydrogels for Cancer Cell Transdifferentiation

**DOI:** 10.34133/research.0810

**Published:** 2025-08-18

**Authors:** Xueqing Ren, Yachao Wang, Mengcheng Lei, Yi Zou, Pengjie Li, Fukang Qi, Jinyun Shi, Han Xie, Mingyu Zhang, Wenhui Wang, Lian Xue, Peng Chen, Bi-Feng Liu, Yiwei Li

**Affiliations:** ^1^ Key Laboratory of Molecular Biophysics of MOE and Hubei Bioinformatics & Molecular Imaging Key Laboratory, Department of Biomedical Engineering, College of Life Science and Technology–Key Laboratory for Biomedical Photonics of MOE at Wuhan National Laboratory for Optoelectronics, Huazhong University of Science and Technology, Wuhan 430074, P. R. China.; ^2^ Henan Key Laboratory of Chronic Disease Prevention and Therapy & Intelligent Health Management, The First Affiliated Hospital of Zhengzhou University, Zhengzhou 450052, P. R. China.

## Abstract

Disrupted matrix mechanics have been found to be highly associated with increased risks of many diseases, including neurodegenerative diseases and cancers. For centuries, the aged tissue matrix has been found to lose its mechanical integrity and exhibit altered biophysical properties. Whether the mechanical properties of matrix serve as a regulator for maintaining the health and function of cells remains unknown. Here, we propose that cells cultured within a tissue-mimicking mechanical microenvironment exhibit reprogrammed cellular behaviors. We first construct a tissue-mimicking hydrogel by combining both viscoelastic and nonlinear elastic components, on which fibroblasts crowd together to form mesenchymal aggregates instead of individually spreading out. The mesenchymal aggregates not only obtain the elevated expression of stemness genes but also exhibit enhanced bidirectional differentiation potentials. The formation of mesenchymal aggregates happens through the reorganization of the collagen network induced by the enhanced cell contraction. Compromising the cell contraction not only prevents the formation of mesenchymal aggregates but also eliminates cell reprogramming. Additionally, this mechanical reprogramming with tissue-mimicking hydrogels has been applied to non-small-lung cancer cells and promotes their adipogenic transdifferentiation, which eventually reverses their epithelial-to-mesenchymal transition genes and suppresses the expression of oncogenes/pro-oncogenes. Thus, our study paves the way for both regenerative medicine and cancer treatments with an approach termed mechanical reprogramming on tissue-mimicking hydrogels.

## Introduction

The native tissue matrix hosts a unique microenvironment for maintaining the homeostatic states of cells, enabling it to be fully functionalized for decades during human life. After being transferred from the native tissue matrix to an in vitro culturing disk, the primary cells rapidly derive from their original states in the tissue, undergoing differentiation or aging and gradually losing their own identities (such as self-renewing, differentiation capabilities, and proliferation). In native tissues, the aging process breaks up the microenvironment of native tissues, including their mechanical integrity, resulting in reduced deformability and a more fibrillar architecture. Along with such biophysical alterations in the extracellular matrix (ECM), the cells obtained features of senescence and compromised functions. More importantly, as a consequence of the cells having an impaired functionality after leaving their original biophysical microenvironment, the risks of many diseases are increased, including those of Alzheimer’s disease, cardiovascular diseases, osteoarthritis, and even cancers.

To treat syndromes from impaired cell functions, a promising therapy is to reprogram the cells (e.g., fibroblasts) and replace the impaired cells with them [1,2]. Until now, the reprogramming of impaired cells has been widely regulated in either biochemical or genetic manner [3–5]. Whether the impaired cells can be directly reprogrammed with the physiologically relevant mechanical cues has not been fully explored [6,7]. Some earlier studies have demonstrated that mechanical cues, such as cell alignment [8], laterally confined culturing [9], 3-dimensional culturing [10], and volumetric compression [11–15], can either partially reprogram the cells or assist in the reprogramming of the cells. A more recent paper demonstrated the development of shell-hardened macroporous hydrogels that provide spatiotemporally programmed mechanical cues, successfully promoting stem cell osteodifferentiation and enhanced bone regeneration [16]. In addition to these mechanical cues, viscoelasticity and nonlinearity have recently been shown to be important physiological characteristics of native tissues, including both aged and young tissues [17,18]. Not only the ECMs within many types of tissues exhibit both viscoelasticity and nonlinear elasticity [17,19–21], but so do cells with interpenetrated cytoskeletons [22–26]. However, the current synthetic or naturally derived hydrogels mainly mimic either viscoelasticity [18,27,28] or nonlinearity [20,21,29], respectively. Despite current advances having revealed the important functions of either viscoelasticity or nonlinearity in tissue regeneration, stem cell differentiation, and tumor progression [28,30–34], whether viscoelasticity and nonlinearity are simultaneously required and working together in native tissues for maintaining cellular functions and youth remains unknown.

Toward this end, we propose to directly mimic native tissue with an interpenetrated hydrogel, termed tissue-mimicking hydrogel, which is composed of one viscoelastic and another nonlinear elastic component, just like the native tissues. Practically, we constructed a tissue-mimicking hydrogel by forming a collagen–alginate interpenetrated hydrogel network. In this interpenetrated network (IPN), collagen contributes to its nonlinearity [35–37], while alginate exhibits a viscous shear-thinning behavior [17,18,38–40]. With such an IPN system, we were able to vary the calcium-based cross-linking between alginate to obtain different initial storage moduli with consistent concentrations of both collagen and alginate. The first intriguing finding is the aggregation of migrative mesenchymal cells via matrix-mediated long-distance cell–cell mechanical interaction; this behavior cannot be observed on either collagen or alginate–RGD alone. The enhanced cell contractility, via a higher initial storage modulus of the hydrogel, promotes cells to crowd together to form aggregates; in comparison, cells spread on a larger area on a stiffer substrate where the substrate is purely elastic (such as polydimethylsiloxane and polyacrylamides) [41–44]. This is also contrary to common sense that cell aggregates form more likely on a nonadhesive substrate. A compromised cell contractility or alteration in collagen mechanics can reverse the cell aggregates back to individually spreading cells. The cross talk between the enhanced contractility and the mechanics of the tissue-mimicking hydrogel is essential in cell aggregation. As a consequence, the formation of mesenchymal aggregates leads to elevated differentiation potentials. The mesenchymal aggregates formed on the tissue-mimicking hydrogel demonstrate elevated expression of pluripotent genes, as well as enhanced capability for both adipogenesis and osteogenesis. This tissue-mimicking hydrogel is also able to reprogram other cells, including cancer cells. Non-small-cell lung carcinoma cells exhibit an obvious mesenchymal–epithelial-transition-like (MET-like) behavior, which enables the cells to easily transdifferentiate into an adipogenic phenotype. By inducing the adipogenesis of cancer cells on the tissue-mimicking hydrogel, oncogenic/pro-oncogenic genes are effectively compromised, which thus suggests potential application of tissue-mimicking hydrogels in cancer transdifferentiation therapy.

## Results

### Establishment and characterization of tissue-mimicking hydrogels

**Fig. 1.**
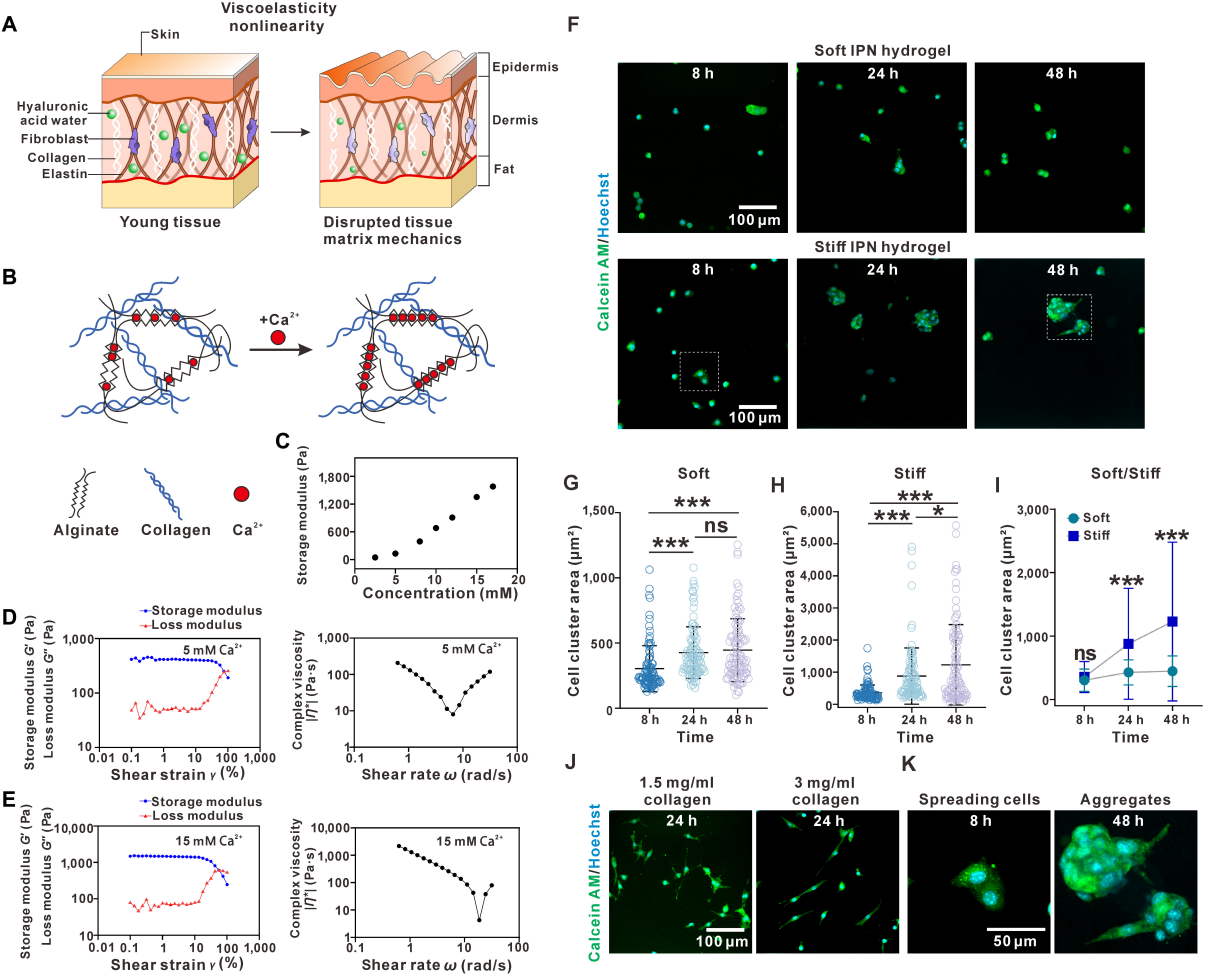
A tissue-mimicking hydrogel with viscoelasticity induced formation of mesenchymal aggregates. (A) Schematic illustrating the different viscoelasticities of young tissues and disrupted tissues. (B) Schematic illustrating alginate–collagen interpenetrated networks (IPNs). (C) The initial storage moduli of IPN hydrogels with various amounts of calcium for cross-linking. (D) Storage and loss moduli as a function of shear strain for soft IPN gels (left); complex viscosity as a function of shear rate for soft IPN gels (right). (E) Storage and loss moduli as a function of shear strain for stiff IPN gels (left); complex viscosity as a function of shear rate for stiff IPN gels (right). (F) Images showing the formation of mesenchymal aggregates on viscoelastic nonlinear IPN hydrogels with different initial elastic moduli (129.43 and 1,352.5 Pa). (G) Cell cluster areas (*n* = 100) of mesenchymal aggregates on soft viscoelastic nonlinear IPN hydrogel at different time points. (H) Cell cluster areas (*n* = 100) of mesenchymal aggregates on stiff viscoelastic nonlinear IPN hydrogels at different time points. (I) Comparison of the cell cluster areas of mesenchymal aggregates between soft and stiff viscoelastic nonlinear IPN hydrogels. (J) Representative images of fibroblasts on pure collagen hydrogels with different concentrations. (K) Representative images of fibroblasts on stiff viscoelastic nonlinear IPN hydrogels at different time points. Data are mean ± SD; *P < 0.05 and ***P < 0.001; ns, not significant.

Recent studies have highlighted the important role of viscoelasticity and nonlinearity in human tissues, which have also been suggested to play an important role in the hemostasis and functionality of native tissues. For instance, rheological testing reveals that both young and aged skin tissues exhibit shear-thinning viscoelastic behavior, with young skin demonstrating a higher storage modulus at the limit of zero strain (Fig. 1A) [45]. Despite hydrogels that mimic either viscoelasticity or nonlinearity having been developed, a hydrogel system that combines the components of both viscoelasticity and nonlinearity has not been fully explored. Moreover, the unique function of a matrix with both components of viscoelasticity and nonlinearity in cells was totally unclear. Toward this end, we employed an alginate–collagen IPN system (Fig. 1B and Fig. S1). In alginate–collagen IPN hydrogels, the alginate hydrogel has been widely used for controlling the stress relaxation behavior as it shows a shear-thinning property [18,28], while the collagen network has been demonstrated to exhibit a nonlinear mechanical behavior [36,37]. To vary the storage modulus of the hydrogel at zero strain for mimicking differentially aged tissue, we supplied the hydrogel with calcium at different concentrations [46]. At the same time, both the concentrations of alginate and collagen were kept the same to maintain their similar stress relaxation and consistent binding sites for cells. Notably, while calcium-cross-linked alginate hydrogels are known to exhibit potential instability in physiological solutions, our interpenetrating network design significantly enhances mechanical stability through the structural support provided by the collagen network. We implemented rigorous standardization protocols for hydrogel preparation, including controlled temperature during gelation (37 °C), precise calcium concentration, and a consistent cross-linking duration (30 min), resulting in highly reproducible mechanical properties. The mechanical stability of our IPNs was verified through extended rheological testing, maintaining consistent viscoelastic properties throughout the experimental timeframe. This enhanced stability and reproducibility enable reliable investigation of mechanically induced cellular reprogramming effects that represent an important innovation over conventional hydrogel systems. Indeed, the storage modulus of the hydrogel at zero strain increased as we increased the calcium concentration in the components (Fig. 1C). The rheological analysis further confirmed the viscoelastic properties of both our stiff and soft IPN hydrogels (Fig. 1D and E and Fig. S1a). Thus, we established an IPN hydrogel system to mimic the mechanical properties of tissues with 15 or 5 mM calcium, 1.5 mg/ml collagen, and 10 mg/ml alginate as components.

### Mesenchymal cells aggregate on the tissue-mimicking hydrogel after their initial spreading

Fibroblasts are one of the major components in connective tissues that reduce proliferative and metabolic activity after disruption of local mechanical features. 3T3-L1 fibroblasts were grown on the tissue-mimicking hydrogel to monitor their cellular behaviors. The fibroblasts were able to spread on the surfaces of both the stiff and soft tissue-mimicking hydrogels (Fig. 1F and H). Surprisingly, after 8 h of culturing, the fibroblasts started to migrate toward each other to form mesenchymal aggregates (Fig. 1F and Movie S1). A higher initial elastic modulus of the tissue-mimicking hydrogel strongly promotes the formation of the aggregates of the mesenchymal cells (Fig. 1F to I). In comparison, the increased stiffness usually promotes cell spreading and prevents cell aggregation on linear elastic substrates such as polydimethylsiloxane (Fig. S2). If we culture fibroblasts on either collagen or alginate substrates alone, the fibroblasts also kept their spreading morphology (Fig. 1J and Fig. S3). Given the fact that some mesenchymal cells were able to form aggregates with their attachment compromised, we carefully monitored their morphological changes after the fibroblasts started to attach to the surface. As we mentioned above, the fibroblasts were indeed able to spread out on the surface of the tissue-mimicking hydrogel (Fig. 1F). As time increased, the fibroblasts started to form small cell aggregates of few cells, and finally, these aggregates fused with each other to form a larger one with a diameter of up to 100 μm (Fig. 1F) with a good cell viability; varied concentrations of calcium to cross-link the IPN hydrogel did not compromise the cell viability of the cultured cells (Fig. S4). With z-stack visualization of the mesenchymal aggregates, we clearly showed the aggregates spreading on the tissue-mimicking IPN hydrogel as shown by their invasive branches (Fig. 1K). In addition, we noticed that the mesenchymal aggregates on a stiff tissue-mimicking hydrogel were larger and exhibited an invasive morphology as compared with those on soft ones (Fig. 1K); this result suggested that the capabilities of cell spreading and aggregation are synergistically promoted on the stiff tissue-mimicking hydrogel, which is different from their antagonistic effect on linear elastic substrates [41,47]. We also varied the concentrations of alginate in IPN hydrogels to adjust mesh density, which showed that a higher mesh density resulted in larger cell aggregates formed (Fig. S5).

### Mesenchymal aggregates induced enhanced collagen reorganization in the presence of the dynamic ionic cross-linking of alginate

**Fig. 2.**
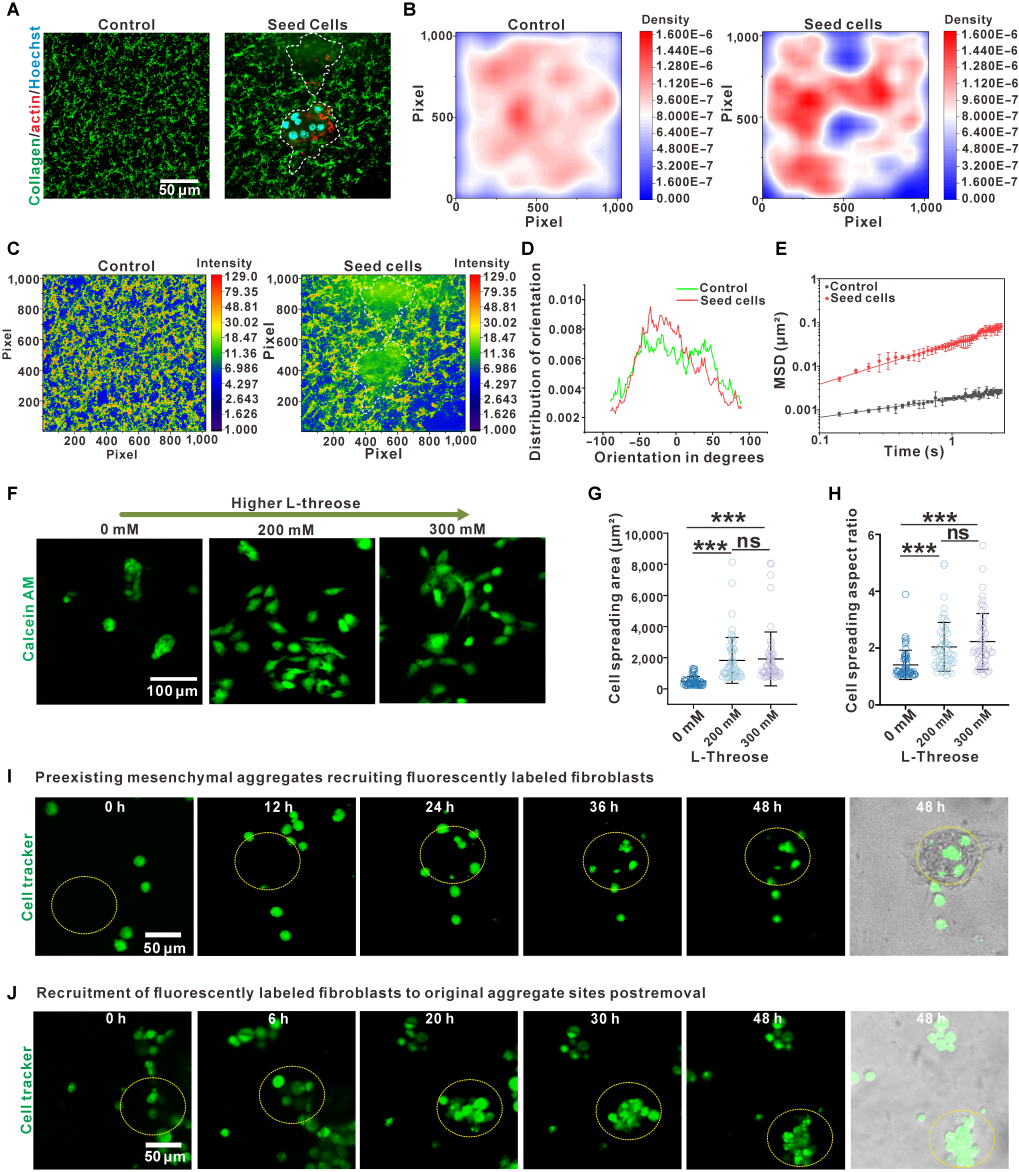
Mesenchymal aggregates induced collagen reorganization in the presence of the dynamic ionic cross-linking of alginate. (A) Fluorescent images showing the redistribution of collagen fibers in the viscoelastic nonlinear IPN hydrogel after the formation of mesenchymal aggregates. (B) Estimated density distribution showing the heterogeneous redistribution of collagen fibers: the collagen fibers were concentrated into certain areas after the formation of mesenchymal aggregates. (C) Heatmaps of fluorescently labeled collagens showing thicker collagen bundle formation. (D) Histograms of collagen fiber orientation showing aligned fibers with mesenchymal aggregates and an isotropic distribution of collagen fibers without cells. (E) Two-dimensional mean square displacement (MSD) of 500-nm tracer particles plotted against lag time on a logarithmic scale in stiff viscoelastic nonlinear IPN hydrogels with or without cells. The viscoelastic nonlinear IPN hydrogel with mesenchymal aggregates exhibited a more diffusive behavior of tracer particles. (F) Fibroblasts spread (*n* = 54) on the viscoelastic nonlinear IPN hydrogel after the collagen fibers were cross-linked by l-threose. (G) When the collagen fibers were cross-linked by l-threose, cell spreading areas increased. (H) When the collagen fibers were cross-linked by l-threose, the cell spreading aspect ratio increased. (I) The preexisting mesenchymal aggregates attracted the newly seeded fluorescently labeled fibroblasts. (J) The position of the preexisting mesenchymal aggregates attracted the newly seeded fluorescently labeled fibroblasts even after removing the mesenchymal aggregates. Data are mean ± SD; ***P < 0.001; ns, not significant.

Since the tissue-mimicking IPN hydrogel induced more obvious aggregation of fibroblasts compared to either a pure elastic substrate or collagen alone, we attributed the formation of the mesenchymal aggregates to the viscoelasticity of the hydrogel (Fig. 1F). Given that the binding sites were provided by collagen in the tissue-mimicking hydrogel, we particularly suggested that the cell–cell attraction happened through the collagen fibers. Therefore, we labeled collagen and analyzed its structure between the groups with or without mesenchymal aggregates. Indeed, the collagen fibers were homogeneously distributed without the cultured cells. In comparison, thicker and more sparse collagen fibers were observed after the formation of mesenchymal aggregates (Fig. 2A); in summary, the overall density of collagen surrounding the formed mesenchymal aggregate was increased. Practically, we quantified the density distribution of collagen network by employing a kernel density estimation, which suggested a 1.34-fold collagen densification, as well as 1.24-fold increments in coefficient of variation (Fig. 2B); this is consistent with a direct measurement of the fluorescent intensity of collagen (Fig. 2C). In addition, the distribution of orientation of the collagen fibers further confirmed the reorganization induced by the mesenchymal aggregates (Fig. 2D). Together, our analysis revealed the formation of collagen bundles and their reorganization upon the formation of mesenchymal aggregates, suggesting possible mechanical reciprocity between the cells and the ECM.

To measure and compare the changes in the moduli of the matrix in situ between those with or without mesenchymal aggregates, we applied passive microrheology with suspended microparticles. The microparticles near the mesenchymal aggregates were not selected for the measurements to avoid matrix fluctuations induced by rhythmic cell contraction. The mean square displacement of 500-nm latex microparticles indicated large thermally driven fluctuations after the mesenchymal aggregates formed, suggesting that the hydrogel underwent a fluidization-like process by the modification of cells (Fig. 2E). The thicker and more dispersed collagen fiber bundle formations resulted in the residual area containing noninterpenetrated/few interpenetrated collagen fibers; this eventually led to these areas decreasing their elastic modulus and exhibiting more viscous behaviors. Given the abovementioned viscoelastic and nonlinear behaviors of the collagen redistribution, we inferred that the viscoelasticity and nonlinearity in the tissue-mimicking hydrogel played a key role in the formation of mesenchymal aggregates.

To test whether inhibition of the reorganization of the hydrogel prevents the formation of mesenchymal aggregates, we cross-linked collagen networks using l-threose while reserving the binding site for cells, which also decreased the viscoelastic relaxation time of collagen as reported [33]. Culturing of fibroblasts led to the spreading of cells on the gel instead of the formation of mesenchymal aggregates (Fig. 2F), as indicated by their larger area (Fig. 2G) and larger aspect ratio (Fig. 2H). We also cross-linked the collagen networks with glutaraldehyde [33], which reduced the hydrogel’s stress relaxation and prevented cell attachment onto the collagen network [48,49]. In this case, the seeded fibroblasts neither spread nor formed mesenchymal aggregate (Fig. S6). Together, we demonstrated that the stress relaxation of the collagen was essential for the induction of the formation of mesenchymal aggregates. Despite the observed reorganization of the collagen bundles in the matrix, whether the modified matrix can directly induce the formation of mesenchymal aggregates was still unclear. To test for this, we seeded the second population of fluorescently labeled fibroblasts onto the matrix with the preexisting mesenchymal aggregates (day 2) and tested whether the newly seeded fibroblasts would migrate toward and engage the preexisting mesenchymal aggregates. Indeed, after 12 h, the majority of the newly seeded fibroblasts engaged with the former mesenchymal aggregates and slowly covered them (<24 h) (Fig. 2I); this suggested that the mesenchymal aggregates serve as an attractor for motile free cells. This was unlike what happened through a chemoattractant since the fibroblasts on neither pure collagen nor a 2-dimensional (2D) flat dish formed such mesenchymal aggregates. Thus, the attractor role of the mesenchymal aggregates was more likely aroused by the reorganization of the matrix. One particular difference in the reorganization of the collagen of our matrix to that of pure collagen was the formation of collagen bundles and their inhomogeneous reorganization. To preserve the reorganization of the collagen but remove the cellular components, we swelled the cells with distilled water and rinsed the tissue-mimicking IPN to remove the preexisting mesenchymal aggregates (Fig. 2J). We again seeded the second population of fluorescently labeled fibroblasts and imaged their behaviors (Fig. 2J). Surprisingly, even without the preexisting cellular components, the fluorescent fibroblasts again move to form aggregates at the location of the former aggregates (Fig. 2J).

### Enhanced contractility is essential for cell aggregation on the tissue-mimicking hydrogel

**Fig. 3.**
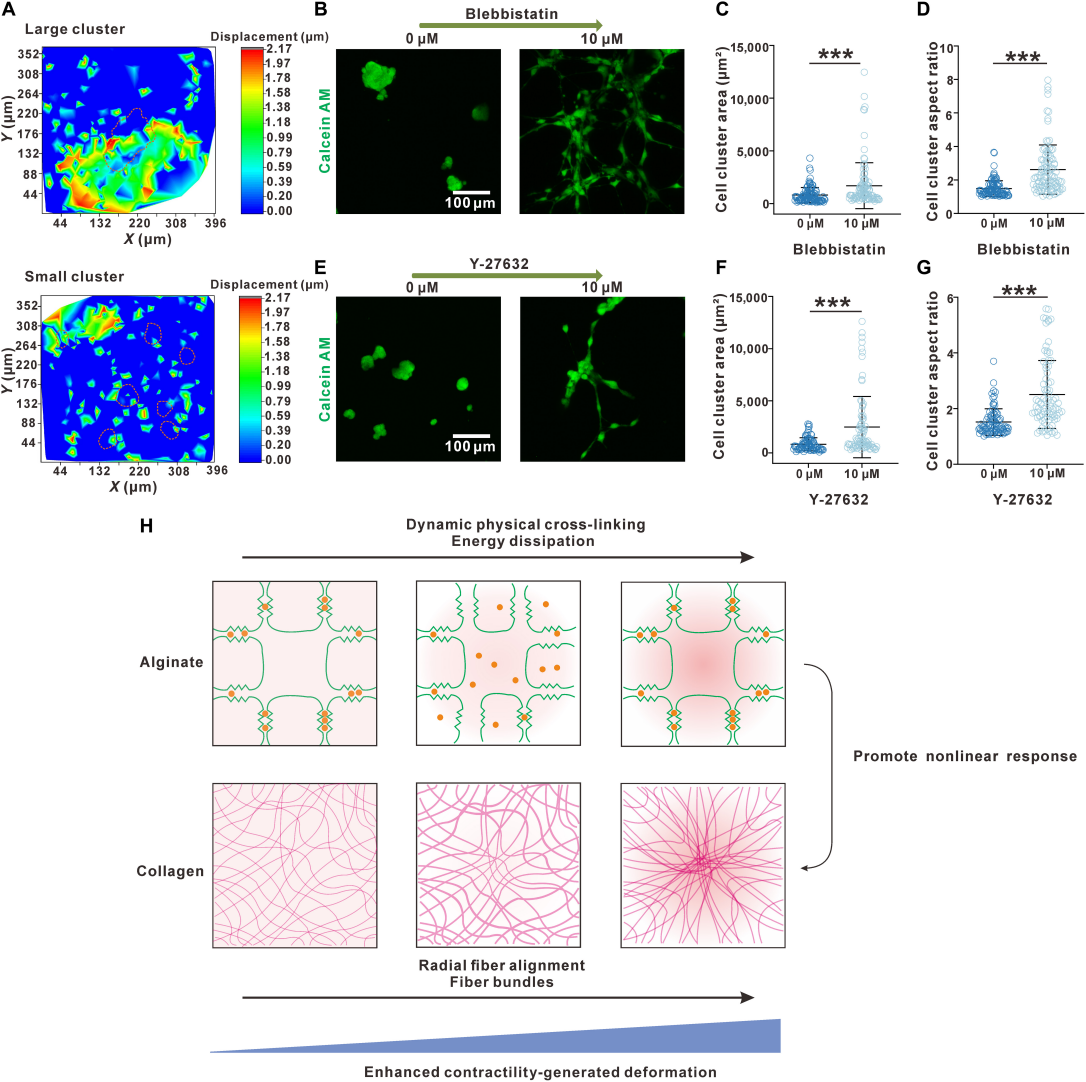
Enhanced contractility is essential for the formation of mesenchymal aggregates on the viscoelastic tissue-mimicking hydrogel. (A) Displacement map of embedded 1-μm tracer particles showing that larger mesenchymal aggregates induced larger deformation on the substrate hydrogel. (B) Inhibition of cell contractility by 10 μM blebbistatin led to cell spreading rather than formation of aggregates without blebbistatin. (C) Cell cluster areas (*n* = 103) increased after treatment with 10 μM blebbistatin. (D) The cell cluster aspect ratio (*n* = 103) increased after treatment with 10 μM blebbistatin. (E) Inhibition of cell contractility via Rho-associated protein kinase (ROCK) by 10 μM Y-27632 led to cell spreading rather than formation of aggregates without blebbistatin. (F) Cell cluster areas (*n* = 86) increased after treatment with 10 μM Y-27632. (G) The cell cluster aspect ratio (*n* = 86) increased after treatment with 10 μM Y-27632. (H) Schematic illustration showing that the dynamic ionic cross-linking of the alginate network promoted the redistribution of collagen fibers. Data are mean ± SD; ***P < 0.001; ns, not significant.

Given the importance of cell–matrix interactions provided in the nonlinear and viscoelastic hydrogel [17], we next examined whether it was the formation of mesenchymal aggregates that actively deformed and modified the mechanical properties of the hydrogel underneath. To do so, fluorescent latex particles (1 μm) were embedded into our tissue-mimicking hydrogel before seeding the cells. By tracking the particles in the hydrogel under the mesenchymal aggregates before and after their formation, we reconstructed a deformation map of the hydrogel induced by the mesenchymal aggregates (Fig. 3A). The larger mesenchymal aggregate induced a larger deformative area on the hydrogel as compared to those smaller ones, which was consistent with the observation that the larger mesenchymal aggregates spread larger areas on the surface. These results also confirmed that the formation of mesenchymal aggregates was not a consequence of cell detachment but, instead, could be attributed to their enhanced interaction and contraction. As reported in previous literature, a stiffer substrate usually leads to stronger cell contractility [41,47], which might be the reason why a stiffer tissue-mimicking hydrogel induces larger mesenchymal aggregates. To further verify whether the formation of mesenchymal aggregates was benefiting from the enhanced cell contractility, we treated the cells with either blebbistatin or Y-27632 to suppress their contraction (Fig. 3B to G). Indeed, when the contractility of cells was reduced, the mesenchymal aggregates dissociated into individually spreading cells (Fig. 3B to G), exhibiting a similar spreading phenotype on stiff linear elastic hydrogels with increased areas and aspect ratios. In comparison, the spreading cells detached from the stiff elastic substrate with treatment with blebbistatin or Y-27632 [50,51]. A more direct piece of evidence is that when we treat the formed mesenchymal aggregates with Y-27632 to inhibit cell contractility, the cells spread and migrated to dissociate the mesenchymal aggregates in a time-course-dependent manner (Fig. S7). Thus, we conclude that the enhanced contractility of the mesenchymal cells on our tissue-mimicking viscoelastic hydrogel is essential for the induced formation of mesenchymal aggregates.

One interesting phenomenon is that both the reorganization of collagen fibers and the formation of mesenchymal aggregates were much more obvious on our tissue-mimicking hydrogel than on pure collagen (Fig. 1F, J, and K). We can argue that it is alginate that assists in the reorganization of the collagen. Indeed, alginate as a component of the IPN hydrogel was reported to contribute to energy dissipation and enhancement of stretchability [52]. In the tissue-mimicking hydrogel, the alginate chains were entangled with the collagen fibers. As the cells increase their contractility and stretch the collagen, the alginate network unzips progressively while the collagen network remains intact. As only the ionic cross-links are broken and the alginate chains themselves remain intact, the ionic cross-links can reform, leading to retaining the stress and strain applied to the collagen. The alginate network works like a mesh that keeps locking and unlocking the collagen fibers. When contraction is applied, alginate unlocks with broken ionic cross-links to allow the deformation of the collagen; at the same time, the alginate network also occasionally locks the deformation of collagen when their ionic cross-links reform. This process repeatedly happens during the migration and aggregations of fibroblasts, which eventually leads to the dramatic reorganization of the collagen networks as we observed (Fig. 3H).

### The reprogramming of fibroblasts on the tissue-mimicking hydrogel elevates their differentiation potentials

**Fig. 4.**
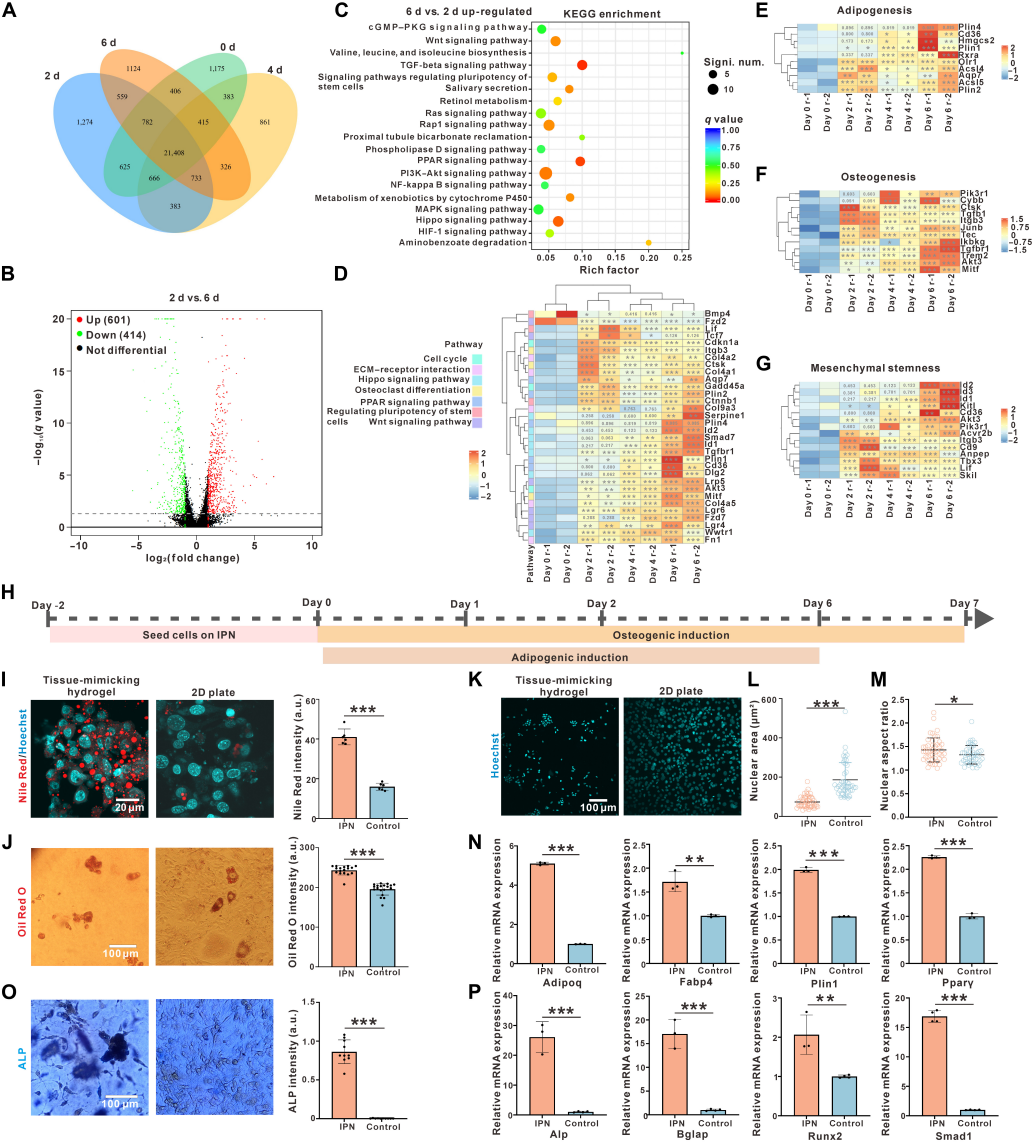
The tissue-mimicking hydrogel induced cell rejuvenation and enhanced bidirectional differentiation potentials. (A) Venn diagram showing the numbers of differentially expressed genes (DEGs) and overlapping genes among the groups of cells cultured for 0, 2, 4, and 6 d. (B) Volcano plot displaying the significance of changes in gene expression between the cells cultured for 2 and 6 d. (C) Bubble plot showing the DEGs’ enriched Kyoto Encyclopedia of Genes and Genomes (KEGG) pathways terms between cells cultured for 2 and 6 d. (D) Heatmap showing the DEGs of cells cultured on hydrogels for different days associated with signaling pathways, including cell cycle, extracellular matrix (ECM)–receptor interaction, the Hippo signaling pathway, osteoclast differentiation, the PPAR signaling pathway, regulation of the pluripotency of stem cells, and the Wnt signaling pathway, according to the KEGG pathway database. (E) Heatmap showing representative DEGs involved in adipogenesis of cells cultured on hydrogels for different days. (F) Heatmap showing representative DEGs involved in the osteogenesis of cells cultured on hydrogels for different days. (G) Heatmap showing representative DEGs involved in the mesenchymal stemness of cells cultured on hydrogels for different days (a Wald test based on a negative binomial generalized linear model [GLM] [*α* = 0.005] analyzed the differential significance (*P* value) of treatment conditions, *n* = 2). (H) Schematic outline of cell rejuvenation and differentiation assays on the viscoelastic tissue-mimicking hydrogel. (I) Cells cultured on the viscoelastic tissue-mimicking hydrogel exhibited elevated adipogenic capability as indicated by Nile Red (*n* = 6). (J) Cells cultured on the viscoelastic tissue-mimicking hydrogel exhibited elevated adipogenic capability as indicated by Oil Red O (*n* ≥ 17). (K to M) Cells cultured on the viscoelastic tissue-mimicking hydrogel exhibited a compressed nucleus (K), as indicated by a decreased nuclear area (L) (*n* = 46) but an increased nuclear aspect ratio (M) (*n* = 46). (N) Quantitative real-time reverse transcription polymerase chain reaction (RT-qPCR) showing the elevated expression of adipogenic genes such as Adipoq, Fabp4, Plin1, and Pparγ on the tissue-mimicking hydrogel as compared to that on a 2-dimensional (2D) collagen-coated culture plate. (O) Cells cultured on the viscoelastic tissue-mimicking hydrogel exhibited elevated osteogenic capability as indicated by ALP (*n*= 10). (P) RT-qPCR showing the elevated expression of osteogenic genes such as Alp, Bglap, Runx2, and Smad1 on that tissue-mimicking hydrogel as compared to that on a 2D collagen-coated culture plate. Signi. num., significant number; mRNA, messenger RNA. Data are mean ± SD; *P < 0.05, **P < 0.01, and ***P < 0.001; ns, not significant.

Mesenchymal cells are known for their isolated, spreading, and migrative behaviors. The loss of their typic features and their transition into epithelial-like cells (adhered to each other and less migrative) are known to be critical for the reprogramming, rejuvenation, and the induction of stemness genes [53,54]. The observed aggregation of fibroblasts on the tissue-mimicking hydrogel made the cells lose their isolated and spreading morphology (Fig. 1F and K). The tight clustering of the fibroblasts in the aggregates also suggested pro-epithelial cell–cell interaction. Thus, we hypothesize that the fibroblasts on the tissue-mimicking hydrogel undergo partial reprogramming. To test for this, transcriptome profiling was carried out on cells cultured on the tissue-mimicking hydrogels at different days (day 0, day 2, day 4, and day 6) (Fig. 4A). When cells on day 6 were compared to those on day 2, 601 up-regulated genes and 414 down-regulated genes were found (Fig. 4B). Kyoto Encyclopedia of Genes and Genomes (KEGG) pathways analysis was performed based on differentially expressed genes (DEGs), showing significant terms between cells cultured for 2 and 6 d (Fig. 4C). To explore the induced molecular processes from the formation of mesenchymal aggregates, we examined several significant DEGs associated with the pluripotency of stem cells, Wnt signaling pathway, TGF-β signaling pathway, PPAR signaling pathway, and Hippo signaling pathway (Fig. 4C). Indeed, genes associated with Wnt signaling and YAP signaling, like Fzd7, Fzd2, Lif, Tcf7, Lgr4, Lrp5, Id2, Id1, Wwtr1, and Akt3, were progressively increased (Fig. 4D). Surprisingly, analysis of DEGs with respect to overrepresented Gene Ontology terms showed up-regulation of processes related to both adipogenesis (genes including Plin1, Plin2, and Aqp7) (Fig. 4E) and osteogenesis (genes including Pik3r1, Trem2, and Akt3) (Fig. 4F), which were usually considered to be mutually suppressed by each other [55,56]; this further turned out to be a result of the elevated mesenchymal stemness (genes including Id1, Id2, Cd36, and Cd9) (Fig. 4G). The observed changes in gene expression were uniquely induced by IPN hydrogels, as we showed that 2D culture conditions generated nonsignificant changes in related genes and pathways (Fig. S8). In addition to the molecular signatures, the cells on the tissue-mimicking hydrogel exhibited an isotropic compressed nucleus (Fig. 4K), as shown by the decreased nuclear area and increased aspect ratio (Fig. 4L and M); this is consistent with previous studies showing an altered nuclear structure during cell reprogramming [52]. Thus, we speculate that the fibroblasts on tissue-mimicking hydrogel undergo partial reprogramming for both enhanced adipogenic and osteogenic potentials.

To test whether the tissue-mimicking hydrogel was able to promote both the adipogenesis and osteogenesis of fibroblasts, after 2 d of culture, fibroblast aggregates were formed on the hydrogel, which were induced to undergo adipogenesis and osteogenesis. After 6 d of the induction of adipogenesis, we compared the oil droplet accumulation between the cells on the tissue-mimicking hydrogel and the cells solely growing on a 2D collagen-coated flask. As indicated by either Nile Red (~2.5-fold increments in signal) or Oil Red O (~1.24-fold increments in signal), the results indicated that there were more lipid droplets accumulated in the fibroblasts with the tissue-mimicking hydrogel as compared to those on the 2D collagen-coated flask (Fig. 4I and J). After 7 d of induction of osteogenesis, we observed a higher expression of ALP in the fibroblasts harvested from the tissue-mimicking hydrogel as compared to that for the 2D flat plate, suggesting their elevated capability of osteogenesis (Fig. 4O). Together, we concluded that the tissue-mimicking hydrogel was able to induce reprogramming of the fibroblasts and elevate their multilineage differentiation potentials. Additionally, quantitative real-time reverse transcription polymerase chain reaction analysis further confirmed that the genes that elevated adipogenic and osteogenic potentials were elevated, such Adipoq, Fabp4, Plin1, Pparγ, Alp, Bglap, Runx2, and Smad1 (Fig. 4N and P).

Moreover, we further investigated the activation of mechanical signaling. KEGG pathway enrichment analysis revealed significant up-regulation of key mechanotransduction pathways in cells cultured on the IPN hydrogel compared to that in day 0 controls, including Hippo signaling, actin cytoskeleton regulation, focal adhesion, and ECM–receptor interaction (Fig. S9a). These pathway-level changes provide strong evidence for the mechanical basis of cellular reprogramming.

Western blot and immunofluorescence analysis confirmed activation of downstream mechanotransduction signaling. Cells showed enhanced cytoskeleton network organization (Fig. S9b) and decreased lamin A/C expression (Fig. S9d). Notably, our hydrogel decreased nuclear YAP accumulation compared to 2D culture (Fig. S9c), indicating that cellular aggregation regulates YAP subcellular localization. This YAP translocation pattern is consistent with reduced commitment to mature differentiated cell fates.

### Enhanced contractility is essential for cell reprogramming on the tissue-mimicking hydrogel

**Fig. 5.**
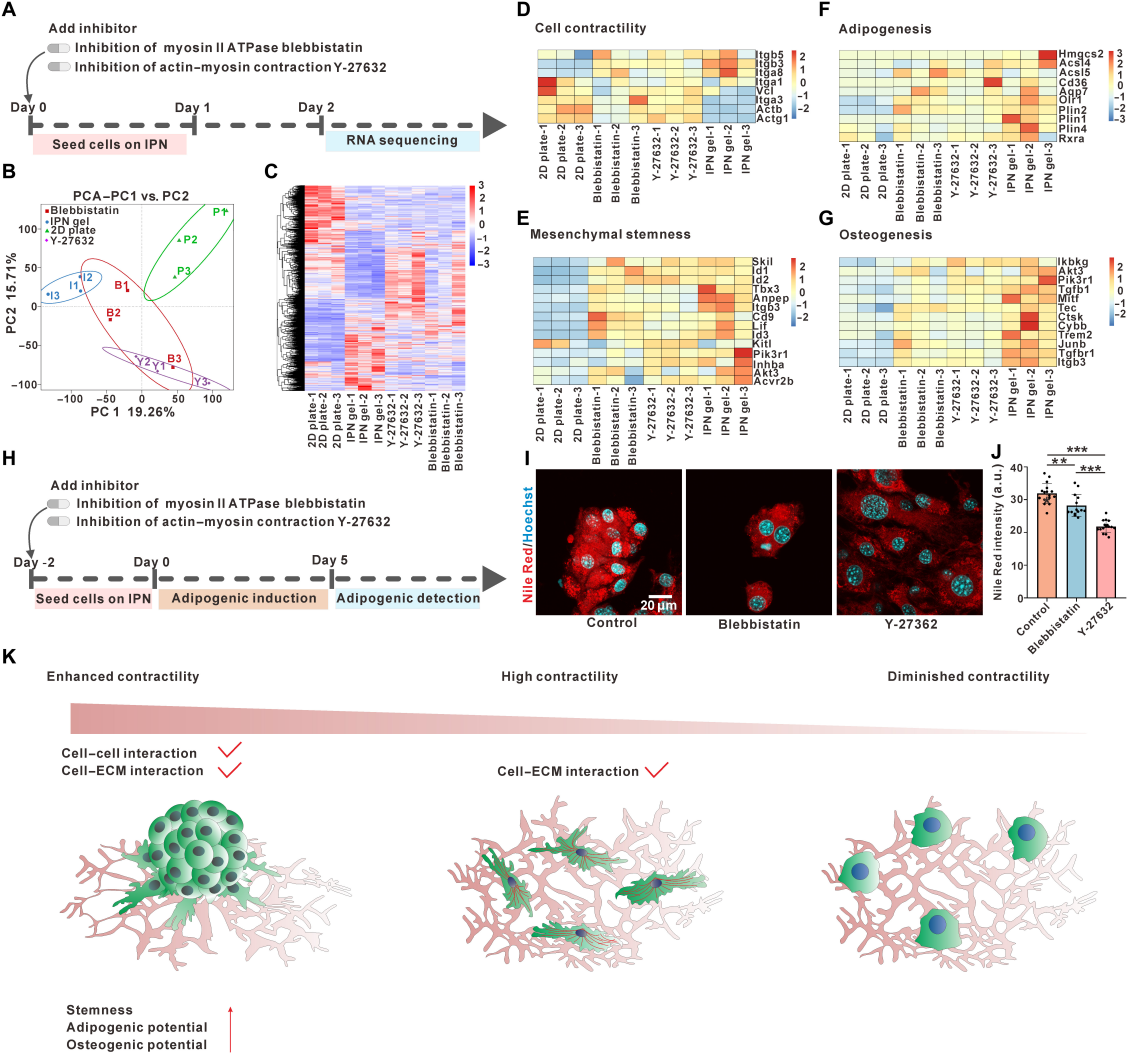
Inhibition of the enhanced contractility of cells on the viscoelastic tissue-mimicking hydrogel prevented cell rejuvenation and enhancement of differentiation potential. (A) Schematic outline of transcriptome profiling for cell rejuvenation under treatment with blebbistatin or Y-27632. (B) Principal component analysis (PCA) plot of groups of cell rejuvenation on either the viscoelastic hydrogel or a 2D collagen-coated plate, with or without treatment with drugs. (C) Differential gene expression clustering heatmap of groups of cell rejuvenation on either the viscoelastic hydrogel or a 2D collagen-coated plate, with or without treatment with drugs. (D to G) Heatmap showing representative genes involved in the cell contractility (D), mesenchymal stemness (E), adipogenesis (F), and osteogenesis (G) of cells cultured on different substrates with or without treatment with drugs. (H) Schematic outline of adipogenic assays for cell rejuvenation under treatment with blebbistatin or Y-27632. (I and J) Accumulation of lipid droplets, as indicated by Nile Red (*n* ≥ 14), showing that inhibition of cell contractility prevented the elevated adipogenesis induced by the viscoelastic hydrogel. (K) Schematic illustration of a working hypothesis: enhanced cell contractility induced the formation of mesenchymal aggregates via the reorganization of the collagen network, which promoted both the cell–cell interaction and cell–ECM interaction, thus eventually elevating stemness and differentiational potentials. Data are mean ± SD; **P < 0.01 and ***P < 0.001; ns, not significant.

Since enhanced contractility has proven to be essential for the formation of mesenchymal aggregates, we speculate that the enhanced contractility of our tissue-mimicking hydrogel is essential for cell reprogramming of fibroblasts. Then, we simply tested whether the fibroblasts’ reprogramming could be prevented by reducing cell contractility. Fibroblasts were seeded onto the tissue-mimicking hydrogel with treatment with either blebbistatin (5 μM) or Y-27632 (5 μM) for a compromised cell contractility (Fig. 5A). After 2 d of culturing, cells were harvested for transcriptome profiling. We compared these cells with drugs to those cultured on either tissue-mimicking IPN hydrogel or a 2D collagen-coated plate without drugs (Fig. 5B and C). Intriguingly, fibroblasts cultured on the tissue-mimicking IPN hydrogel and 2D collagen-coated plate exhibited distinct cell adhesion and contractility patterns. The cells on the tissue-mimicking hydrogel preferred to express Itgb3, Itgb5, Itga8, and Itga1, while the cells on the 2D collagen-coated plate were prone to express Itga3, Vcl, Actb, Actg1, and Itga1; only Itga1 was highly expressed in both conditions. When treated with blebbistatin or Y-27632, the cells on the tissue-mimicking hydrogel started to express Itga3, Vcl, Actb, and Actg1, just like the cells on the 2D collagen-coated plate (Fig. 5D), while the expression of other cell adhesion genes (Itgb3, Itgb5, and Itga8) was suppressed (Fig. 5D). Thus, the cell adhesion on the tissue-mimicking hydrogel was more like that on the 2D collagen-coated plate after the inhibition of cell contractility with either blebbistatin or Y-27632. The same trends were found when we compared the genes regulating pluripotency or differentiation (Fig. 5E to G). The elevated genes associated with mesenchymal stemness (Fig. 5E), adipogenesis (Fig. 5F), or osteogenesis (Fig. 5G) from the priming of the viscoelastic tissue-mimicking hydrogel were again suppressed when cell contractility was inhibited, exhibiting an intermediate state between the 2D collagen-coated plate and tissue-mimicking hydrogels.

To further confirm whether enhanced cell contractility on the tissue-mimicking hydrogel is required for reprogramming and enhanced differentiation capability, we induced the adipogenesis of cells on the hydrogel with or without drugs (blebbistatin or Y-27632) (Fig. 5H). Inhibition of cell contractility indeed led to reduced adipogenic efficiency (Fig. 5I and J), which is consistent with the sequencing result. As noted, treatment with blebbistatin/Y-27632 promotes the adipogenesis of fibroblasts/mesenchymal stem cells when cultured on a 2D collagen-coated elastic or rigid substrate [57–59]. These controversial observations further highlighted the unique impact of viscoelasticity and nonlinear deformation on cell reprogramming and confirmed the essential role of the enhanced contractility during mechanical cell reprogramming (Fig. 5K).

### The reprogramming of cancer cells on the tissue-mimicking hydrogel enhanced their adipogenic transdifferentiation

In addition, cancer is also tightly interconnected with disrupted mechanical features of the matrix [60]. Previous studies hold that aging microenvironments with dysregulated mechanical cues favor the growth and progress of tumors [61,62], among which the altered mechanics and fragmented fibrils in an aged ECM have been reported in recent years to assist tumor development [63,64]. Despite many pieces of evidence showing that an aged matrix with disrupted mechanics assisted tumor progression, whether a hydrogel that mimics the tissue with both viscoelastic and nonlinear components can reverse/the progressed cancer cells and help restrain the development of tumor remains unknown.

Since cell senescence is associated with cancer progression [60,65], whether the observed cell reprogramming on our tissue-mimicking hydrogel can benefit cancer treatment is worth further exploring. In recent years, transdifferentiation therapy has been suggested to be a new potential treatment for cancers [66,67], which requires to induce transdifferentiation of cancer cells into other types of nonproliferative cells such as adipocytes [68–70]. These studies started with epithelial typic cancer cells, which were treated with genetic and biochemical regulators, which usually induced epithelial-to-mesenchymal transition (EMT) on epithelial typic cancer cells. However, the EMT processes of cancer cells were reported to lead to a more malignant phenotype. Thus, the adipogenic transdifferentiation of epithelial typic cancer cells might also accompany the risk of generating malignant cancer cells. Whether adipogenic transdifferentiation can be induced in mesenchymal typic cancer cells is worth being further explored. Additionally, mechanically induced adipogenic transdifferentiation of cancer cells has not been reported.

**Fig. 6.**
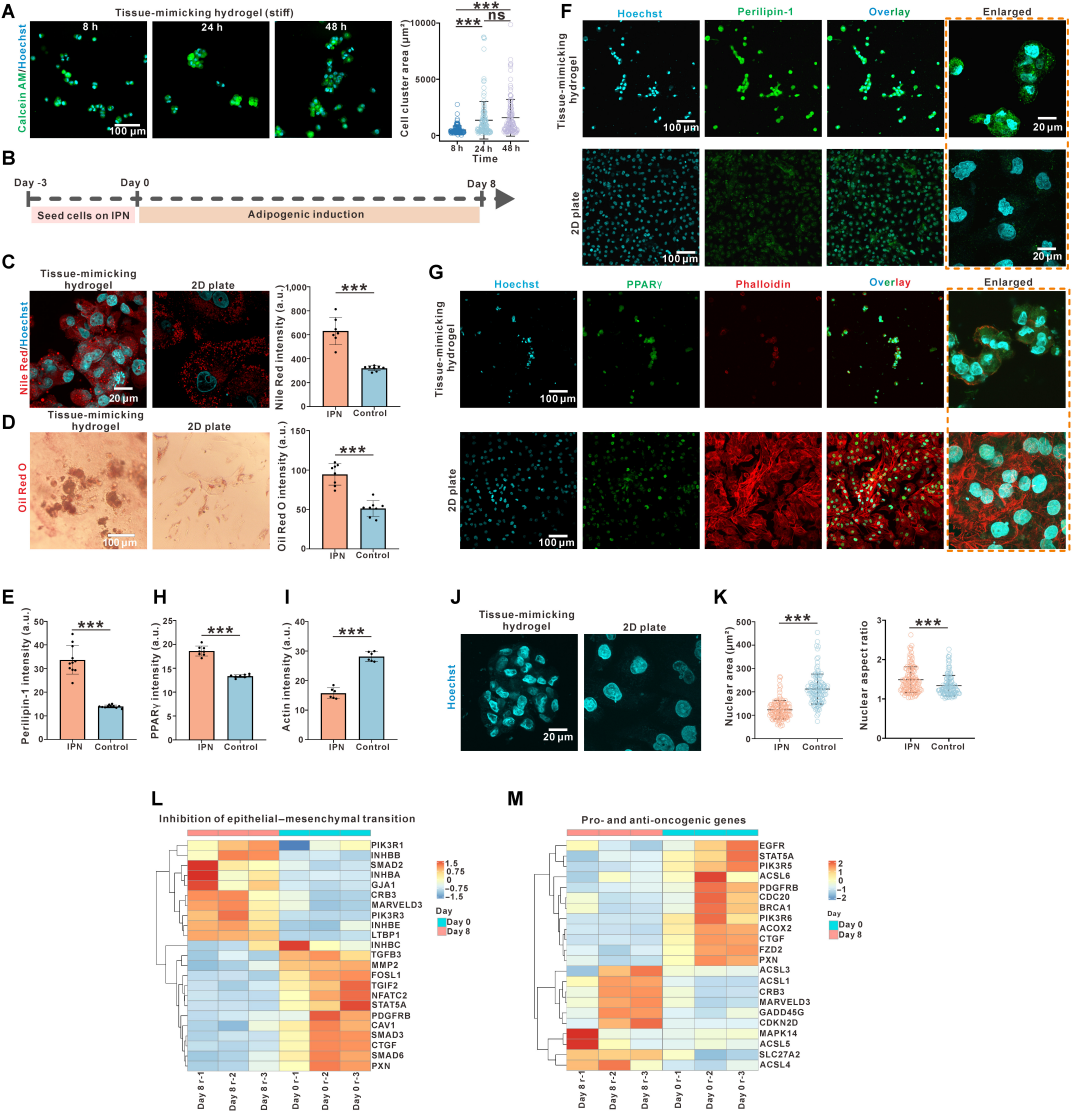
The viscoelastic tissue-mimicking hydrogel was able to enhance the adipogenic transdifferentiation of non-small-cell lung cancer cells. (A) Cancer cells formed mesenchymal aggregates on the stiff viscoelastic tissue-mimicking hydrogel (*n* = 118). (B) Schematic outline of the adipogenic transdifferentiation of cancer cells on the viscoelastic tissue-mimicking hydrogel. (C) Cancer cells cultured on the viscoelastic tissue-mimicking hydrogel exhibited elevated adipogenic transdifferentiation as indicated by Nile Red (*n* ≥ 7). (D) Cells cultured on the viscoelastic tissue-mimicking hydrogel exhibited elevated adipogenic transdifferentiation as indicated by Oil Red O (*n* = 8). (E to I) Immunofluorescent staining showing the elevated expression of adipogenic protein perilipin-1 (*n* = 11) (E and F) and PPARγ (*n* = 8) (G and H), as well as a disrupted actin (*n* = 6) cytoskeleton network (G and I), in cancer cells on the viscoelastic tissue-mimicking hydrogel as compared to that in a 2D collagen-coated culture plate. (J and K) Cells cultured on the viscoelastic tissue-mimicking hydrogel exhibited a compressed nucleus (J), as indicated by a decreased nuclear area (*n* = 125) but an increased nuclear aspect ratio (*n* = 125) (K). (L) Heatmap showing that the cancer cells on the hydrogel expressed genes inhibiting the epithelial-to-mesenchymal transition (EMT) process and genes promoting the mesenchymal–epithelial transition (MET) process. (M) Heatmap showing that the cancer cells on the hydrogel exhibited suppressed the expression of oncogenes/pro-oncogenes and elevated the expression of tumor suppressor genes. Data are mean ± SD; ***P < 0.001; ns, not significant.

Given the fact that our tissue-mimicking hydrogel was able to induce the reprogramming of mesenchymal cells, our matrix with both viscoelastic and nonlinear components could be a possible platform for inducing the adipogenesis of cancer cells in a more invasive state as compared to previously reported cases [69,70]. To test our hypothesis, non-small-cell lung carcinoma H1975 cells were selected for the tested system for their invasive behavior in a partial-EMT state. Indeed, H1975 cells formed aggregates on our tissue-mimicking hydrogel with increasing stiffness (Fig. 6A), despite their spreading behaviors on substrate with linear elasticity. H1975 cells were cultured on the tissue-mimicking hydrogel for 3 d before the induction of adipogenesis. The efficiency of adipogenesis of the cancer cells was examined after 8 d of induction (Fig. 6B). The generation of adipocytes, as assessed by visualizing lipid droplets of fluorescent Nile Red and colorimetric Oil Red, was readily detected in the H1975 cells on the tissue-mimicking hydrogel but not in those on a 2D collagen-coated flask with induction (Fig. 6C and D). Additionally, the protein level expression of perilipin-1 (Fig. 6E and F) and PPARγ (Fig. 6G and H) became obvious in cells after being cultured on the tissue-mimicking hydrogel. Along with acquiring adipocyte-specific features, transdifferentiated H1975 cells lose their mesenchymal, invasive traits. Visualization of the actin cytoskeleton by phalloidin staining revealed a reorganization of mesenchymal stress fibers into cortical actin, a hallmark of cell immobilization (Fig. 6G and I). In addition, the morphology of the nucleus has been recognized as a sign of cell reprogramming, due to its impact on chromatin condensation and accessibility [8,71,72]. Our analysis clearly showed that a deformed and shrank nucleus in the cancer cells on the tissue-mimicking hydrogel, instead of a round and large morphology in those on 2D collagen-coated substrate (Fig. 6J and K).

Furthermore, transcriptional profiling confirmed the successful partial reprogramming and inhibition of cancer progress in our treated non-small-lung cancer cells. One of the significant features is the inhibition of EMT and the induction of MET (Fig. 6L). The MET process has long been recognized as an early signature event during the reprogramming and differentiation of mesenchymal cells [53,54]. The mesenchymal cells that undergo MET start to gain pluripotency and multilineage differentiation potentials. At the same time, the reversion of the EMT process in cancer cells has been suggested to be a possible target for stopping tumor invasion and malignance. Together, the up-regulated genes inhibiting EMT and the down-regulated genes that promote EMT in our system not only indicated the successful reprogramming of cancer cells but also suggested potential inhibition of cancer invasion (Fig. 6L). By KEGG pathway enrichment analysis of DEGs, we obtained altered KEGG terms including TGF-β signaling pathway (regulating EMT process), Adipocytokine signaling pathway (implying adipogenic behavior), gap junction (cell–cell connection change during EMT/MET process), and adherens junction (cell–matrix interaction change during the EMT/MET process) (Fig. S10a and b). Moreover, western blot analysis demonstrated that the cancers after being cultured on tissue-mimicking IPN hydrogels exhibited elevated expression of E-cadherin, decreased expression of vimentin and Snail/Slug; the results together supported the inhibition of EMT and induction of MET biomolecular features in cancer cells on tissue-mimicking IPN hydrogels (Fig. S10c).

For a more direct analysis of cancer progression, we presented the changes in the expression of oncogenic or pro-oncogenic genes before and after cancer transdifferentiation on the tissue-mimicking hydrogel. Consistently, both the oncogenes (EGFR, BRCA1, CDC20, etc.) [73–75] and the pro-oncogenic genes (PIK3R6, PXN, FZD2, etc.) [76–78] were inhibited, while the genes (ACSL1, GADD45G, CRB3, etc.) that inhibit tumor progression [79–81] were up-regulated (Fig. 6M). Conclusively, our evidence supported that the tissue-mimicking hydrogel provided a new way for cancer transdifferentiation therapy via mechanical cell reprogramming.

While our study demonstrated mechanical cell reprogramming and cancer transdifferentiation in vitro, translation to therapeutic applications requires in vivo validation. The physiological environment presents challenges including immune responses, vascularization, and tissue integration that could influence therapeutic outcomes. Future studies will require tumor xenograft models and aged tissue repair models to evaluate therapeutic efficacy and safety profiles. Although the complexity of in vivo validation necessitates follow-up research beyond the current study, our mechanistic insights into matrix-mediated cellular reprogramming provide a foundation for developing therapeutic strategies that harness mechanical cues for regenerative medicine and cancer treatment.

## Conclusion

It has long been recognized that the mechanical properties of human tissues are essential for the maintenance of hemostasis and functionality of healthy tissues. In contrast, an aging matrix with altered mechanical properties has been reported to be associated with diseases for several centuries. Until now, little has been known about whether mechanical homeostasis holds the secret of keeping cells healthy and functionalized. The key innovation of this research lies in the simultaneous integration of both viscoelastic and nonlinear elastic components within a single hydrogel system to mimic native tissue mechanics, but not either alone. While previous studies have investigated either viscoelasticity or nonlinearity separately, our tissue-mimicking approach combines both properties through an alginate–collagen IPN system that recapitulates the complex mechanical environment of natural tissues. In this paper, we first show that a tissue-mimicking matrix, which is composed of both viscoelastic and nonlinear components similar to those of native tissues, could induce the cell reprogramming of both fibroblasts and cancer cells. Cell reprogramming has recently been recognized as an important approach for not only increasing healthspan but also disease treatment. Unlike genetic editing and stem cell replacement that current scientists have widely investigated, our mechanical cell reprogramming relied on the mechanical cues mimicking the tissue matrix, which avoids the possible by-effects, such as off-target effects and formation of teratoma.

Mechanistically, we argue that the underlying mechanism of cell reprogramming is matrix-mediated long-distance cell–cell mechanical cross talk. Indeed, the biophysical reprogramming of cells has been reported, including 3-dimensional encapsulation [10], volumetric compression [11,12], laterally confined culturing [9], and aligned culturing [8]. Consistent with this study, all of these papers showed a crowded growth of mesenchymal cells during their reprogramming, which suggested that establishing enough cell–cell contact is essential for reprogramming mesenchymal cells. This biophysical observation coincidently matches previous genetic findings that MET is required for reprogramming fibroblasts [53,54]. Unlike the abovementioned studies, we show mechanistic discovery of matrix-mediated long-distance cell–cell interactions—the unique phenomenon where cells aggregate through collagen reorganization assisted by alginate’s dynamic cross-linking, which has not been reported with single-component hydrogels. Our result confirmed that the mechanical cues raised from native tissues were sufficient to induce cell reprogramming without genetic manipulation, achieving enhanced stemness and bidirectional differentiation through physical cues.

The key features of the tissue-mimicking matrix in cell reprogramming were the viscoelastic component and nonlinear elastic component, which originated from the reorganization of the collagen fibers and dynamic ion-cross-linking of alginate; each of them (viscoelasticity and nonlinearity) was recently highlighted separately for its unique role in regulating cell behaviors and functions [33,34]. Besides all of the advances, few research studies highlighted that the simultaneous presence of both viscoelastic and nonlinear components is important for cell functions. In this paper, our study provided physiological and functional evidence to support the importance of the recently reported unique cell migration behaviors on viscoelastic hydrogels [33,34,39], linking the biophysical behavior (migration) to cellular function (reprogramming and transdifferentiation). Our study also showed a good example that mesenchymal cells can interact with each other starting from a long distance via a purely mechanical interaction from the ECM.

**Fig. 7.**
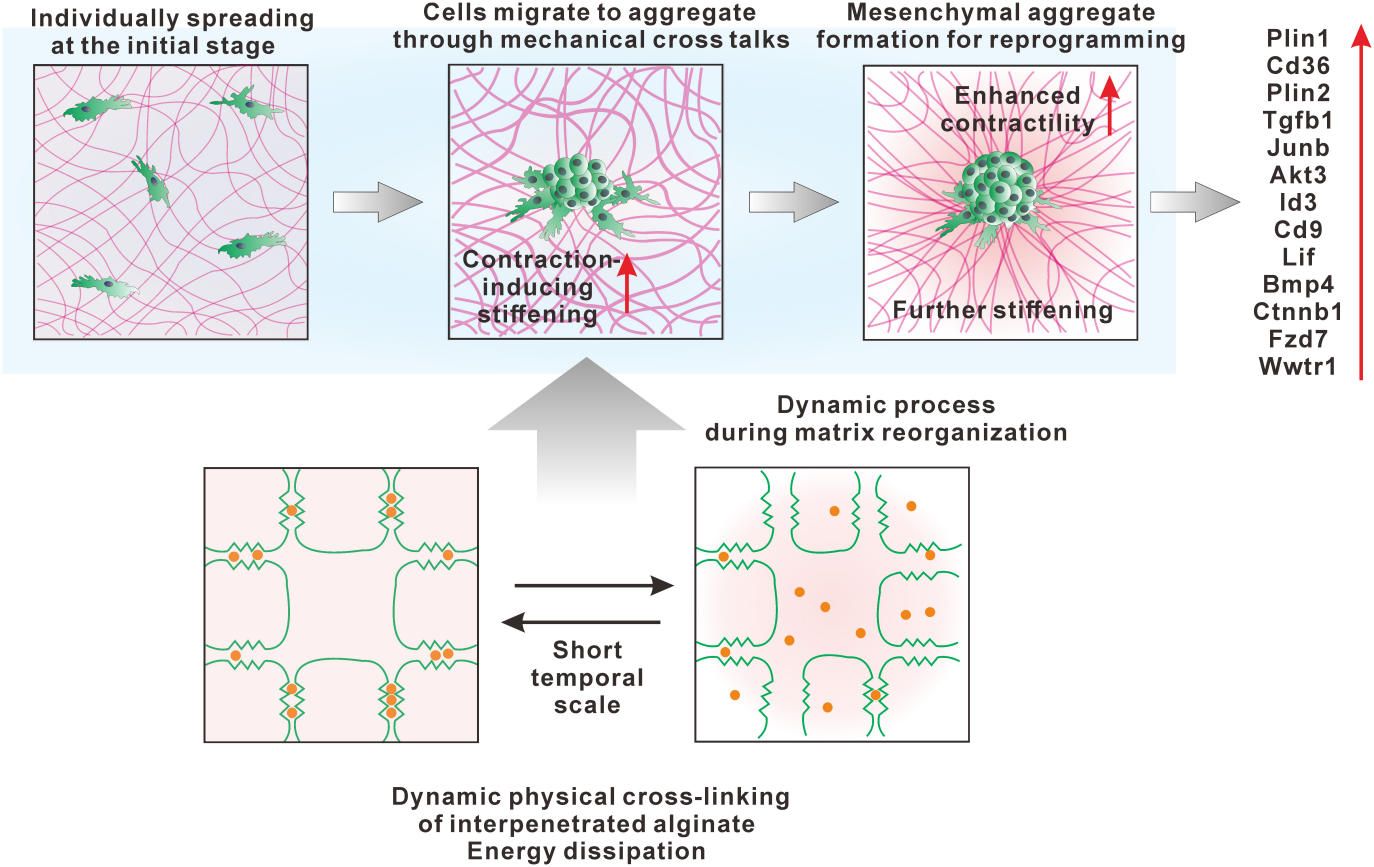
Schematic illustrating the process of how cells cross talk with matrix reorganization. Cells initially spread on the surface of the tissue-mimicking IPN hydrogel. Cellular contraction induces recruitment of collagen fibers, which stiffens the matrix; during this process, the dynamic physical cross-linking of the interpenetrated alginate network conserves the deformation of collagen fiber and enhances matrix reorganization. The increased stiffness of the matrix further induces higher cellular contractility. The feedback loop between the matrix and cell contractility drives cell–cell long-distance cross talk and further induces aggregation of mesenchymal cells on the matrix. As a consequence, aggregation of mesenchymal cells induces the reprogramming of cells with enhanced expression of genes related to stemness, adipogenesis, and osteogenesis.

Mechanistically, our study provides several key insights into the fundamental principles governing cell–matrix interactions in this reprogramming system (Fig. 7). First, we demonstrate a reciprocal mechanosensing loop between cells and the matrix: cellular contractility reorganizes the tissue-mimicking matrix, which in turn modulates cell fate through altered mechanical feedback. Second, we establish that this mechanosensing functions differently on our viscoelastic–nonlinear platform compared to purely elastic substrates. Specifically, the dynamic ion-cross-linking of alginate allows local stress relaxation while maintaining global matrix integrity, creating a unique mechanical niche that supports mesenchymal aggregate formation. Third, we identify a temporal sequence in the mechanotransduction pathway, where initial contractility-driven matrix reorganization precedes transcriptional changes in stemness genes and differentiation pathways. These insights extend beyond the specific materials used in our system and establish broader principles applicable to understanding how cells interpret complex mechanical environments. Furthermore, our identified mechanistic pathway links existing knowledge from disparate fields—material mechanics, cell contractility, nuclear mechanotransduction, and epigenetic regulation—providing a more unified model of how physical cues can direct cell fate determination. This systematic mechanistic framework not only explains our observed phenomena but also provides predictive principles for designing future biomaterial systems for controlled cell reprogramming.

Surprisingly, mechanical reprogramming can also be applied to cancer cells. The mechanical reprogramming of cancer cells increases their plasticity and enables their transdifferentiation into adipogenic cells with diminished proliferation and suppressed oncogenes. Transdifferentiation of cancer cells into nonproliferative or nonmigrative cells has recently been considered as novel cancer treatment, termed transdifferentiation therapy. The transdifferentiation of cancer cells into neuronal cells, neuroendocrine cells, vasculogenic mimicry, and adipocytes has recently been achieved via genetic manipulation [66,67]. For the first time, we demonstrated that our system provided a new mechanical/physical approach for transdifferentiation therapy of tumors. Mechanistically, this is achieved via the induction of MET, which is not only a reverse process of EMT that possibly leads to tumor malignancy [82,83] but also an essential role in cell reprogramming [53,54]. Given the good compatibility of mechanical treatment with biochemical/genetic cocktails for cell transdifferentiation, our mechanical approach has the opportunity to find its new application in other transdifferentiations (neuronal cells or vasculogenic mimicry) of cancer cells beyond adipocyte-like cells in future studies.

The most immediate application lies in developing enhanced cell culture platforms for regenerative medicine. Our hydrogel can serve as an improved substrate for expanding patient-derived cells before therapeutic transplantation, given the demonstrated ability to enhance stemness markers and differentiation potential of fibroblasts. The system could also be developed into injectable scaffolds for tissue repair applications where enhanced cellular aggregation is beneficial. Our findings also provide a valuable research platform for cancer biology studies. The ability to mechanically induce cancer cell transdifferentiation offers a new experimental model for understanding cancer cell plasticity and testing differentiation-based therapeutic strategies. The demonstrated suppression of oncogenes and EMT reversal in lung cancer cells makes this an excellent tool for screening potential treatments. Our approach demonstrates a design principle for creating biomaterials that combine viscoelastic and nonlinear properties to guide cellular behavior. This concept can be extended to develop specialized culture substrates or more sophisticated tissue engineering scaffolds that better recapitulate native tissue mechanics.

While our study demonstrates the potential of mechanical cell reprogramming using tissue-mimicking hydrogels, several limitations warrant consideration. Cell response variability may arise from differences in cell passage numbers, donor characteristics, and culture conditions, which could affect the reproducibility of mechanical reprogramming across different cell populations. Additionally, our alginate–collagen hydrogel system, while effective for fibroblasts and lung cancer cells, may require optimization for other cell types given the diverse mechanical properties of native tissues. Furthermore, while our data strongly support enhanced contractility as the primary driver of cellular aggregation and reprogramming, alternative mechanisms such as altered cell adhesion kinetics, membrane tension changes, or modified paracrine signaling may also contribute to the observed phenotypes. Long-term studies examining cellular behavior over extended culture periods, along with investigations into the hydrogel’s adaptability across different tissue types and disease states, will be essential for advancing this mechanical reprogramming approach toward clinical applications. Future work should also address scalability, standardization protocols, and the integration of additional biological cues to enhance the therapeutic potential of this platform.

Overall, this study exhibited the unique biophysical behaviors of cells on tissue-mimicking hydrogel and highlighted long-distance cell–cell mechanical interaction via the matrix, which significantly affected the cell differentiation potentials in both healthy fibroblasts and cancer cells. The interaction between the cells and matrix can be further viewed as a novel therapeutic target for either regeneration or cancer treatment.

## Methods

Detailed information about relevant materials and methods for this research can be found in the Supplementary Materials.

## Data Availability

The data that support the findings of this study are available from the corresponding authors upon reasonable request.

## Supplementary Materials

Supplementary 1 Resources Table Materials and Methods Figs. S1 to S10 Movies S1 and S2
