# Supplementary material for: Mechanical Cell Reprogramming on Tissue-Mimicking Hydrogels for Cancer Cell Transdifferentiation
Source: Research (Wash D C). 2025 Aug 18;8:0810. doi: 10.34133/research.0810 (PMC12358751; doi:10.34133/research.0810)
Supplement: Supplementary 1 — Resources Table Materials and Methods Figs. S1 to S10 Movies S1 and S2 [file research.0810.f1.zip › SI-Cell Nonlinear-Research-proof.docx]

Supplementary Materials for

Mechanical Cell Reprogramming on Tissue-Mimicking Hydrogels for Cancer Cell Transdifferentiation

**Authors:** Xueqing Ren^1^†, Yachao Wang^2^†, Mengcheng Lei^1^, Yi Zou^1^, Pengjie Li^1^, Fukang Qi^1^, Jinyun Shi^1^, Han Xie^1^, Mingyu Zhang^1^, Wenhui Wang^1^, Lian Xue^1^, Peng Chen^1^, Bi-Feng Liu^1*^, Yiwei Li^1*^

*Correspondence to: yiweili@hust.edu.cn (Y.L.); bfliu@mail.hust.edu.cn (B.-F.L.);

**This PDF file includes:**

Resource table

Figs. S1 to S10

Movie S1

Movie S2

**TABLE S1**

| REAGENT or RESOURCE | SOURCE | IDENTIFIER |
| --- | --- | --- |
| Antibodies |  |  |
| anti-PPARγ Rabbit Monoclonal Antibody | Cell Signaling  Technology | 2443s |
| anti-Perilipin-1 Rabbit Monoclonal Antibody | Cell Signaling  Technology | 9349s |
| anti-Collagen, TypeI, Monoclonal antibody | Sigma | C2456 |
| Rabbit anti-alpha-SMA Polyclonal Antibody | Absin | 130621 |
| anti -YAP Rabbit Monoclonal Antibody | Cell Signaling  Technology | 14074 |
| Mouse anti-Lamin A/C Monoclonal Antibody | Absin | 5D12 |
| anti-E-Cadherin Rabbit Monoclonal Antibody | Cell Signaling  Technology | 24E10 |
| anti-Vimentin Polyclonal antibody | Proteintech | 10366-1-AP |
| anti-Snail/Slug polyclonal antibody | Proteintech | 12129-1-AP |
| anti-Histone H3 Polyclonal antibody | Proteintech | 17168-1-AP |
| anti-GAPDH Monoclonal antibody | Proteintech | 60004-1-Ig |
| HRP-conjugated Goat anti-Rabbit IgG(H+L) | Proteintech | SA00001-2 |
| HRP-conjugated Goat anti-Mouse IgG(H+L) | Proteintech | SA00001-1 |
| Alexa Fluor 488 Goat anti-Rabbit IgG (H+L) | Beyotime Biotechnology | A0423 |
| Dylight 488 Goat anti-Mouse IgG | Abbkine | A23210 |
| Chemicals and Dye |  |  |
| RIPA buffer | Solarbio | R0010 |
| Phenylmethylsulfonyl fluoride (PMSF) | Beyotime Biotechnology | ST505 |
| Bicinchoninic acid protein assay kit (BCA kit) | Beyotime Biotechnology | P0009 |
| Sodium dodecyl sulfate polyacrylamide (SDS–PAGE) | Beyotime Biotechnology | P0012A |
| Polyvinylidene difluoride membrane (PVDF Membrane) | MerckMillipore | IPVH00010 |
| BeyoECL Plus | Beyotime Biotechnology | P0018S |
| Actin-Tracker Red-Rhodamine | Beyotime Biotechnology | C2207S |
| Calcein AM | Beyotime Biotechnology | C2012 |
| Propidium Iodide | Beyotime Biotechnology | ST511 |
| Cell Tracker Green CMFDA(Cell tracker) | Invitrogen | C2925 |
| Latex beads, carboxylate-modified  polystyrene, fluorescent orange | Sigma | L5530 |
| Latex beads amine-modified polystyrene, fluorescent orange | Sigma | L9654 |
| Nile Red | Macklin | N815046 |
| Oil Red | Solarbio | O8010 |
| Hoechst 33258 | Beyotime Biotechnology | C1017 |
| Fast Blue BB Salt hemi(zinc chloride) salt | Sigma | F3378 |
| Naphthol AX-MX Phosphate | Sigma | N4875 |
| IBMX | Sigma | I5879 |
| Insulin | Beyotime Biotechnology | R3375 |
| Dexamethasone | Sigma | D1756 |
| Rosiglitazone | Sigma | R2408 |
| L-ascorbic acid | Sigma | A4544 |
| β-Glycerophosphate | Macklin | D859328 |
| Tris-HCl | Aladdin | T105289 |
| Triton-X-100 | Biosharp | BS084 |
| BSA | Solarbio | A8020 |
| Y-27632 | Santa Cruz  Biotechnology | SC-3536 |
| Blebbistatin | Aladdin | B129580 |
| 10×PBS | Biosharp | BL315A |
| NAOH | Sinopharm Chemical Reagent Co.,Ltd | 10019762 |
| Paraformaldehyde (PFA) | Biosharp | BL539A |
| Isopropyl alcohol | Macklin | I811925 |
| Trizol | Invitrogen | 15596026 |
| Trichloromethane | Chron Chemicals | 67-66-3 |
| Ethanol | Sinopharm Chemical Reagent Co.,Ltd | 1009218 |
| DMSO | Sigma | D2650 |
| L-Threose | Shanghai yuanyeBio-Technology Co.,Ltd | S52084 |
| NaCl | Sinopharm Chemical Reagent Co.,Ltd | 10019318 |
| Tween-20 | Sinopharm Chemical Reagent Co.,Ltd | 30189328 |
| MgCl_2_ | Sinopharm Chemical Reagent Co.,Ltd | 10012818 |
| CaSO_4_ | Sigma | 255548 |
| Rat Tail Collagen-I | Corning | 354236 |
| Sodium Alginate | Aladdin | S100127 |
| Polydimethylsiloxan (PDMS) | Dow Corning | 1673921 |
| GGGGRGDSP | Xi’an ruixi  Biologoical  Technology  Co.,Ltd |  |
| C_4_H_4_NNaO_6_S | Macklin | N875148 |
| C_8_H_18_CIN_3_ | Macklin | N808856 |
| C_6_H_13_NO_4_S | Macklin | M5159 |
| All-in-one RT SuperMix perfect for qPCR | Vazyme | R333-01 |
| Taq Pro Universal SYBR qPCR Master Mix | Vazyme | Q712-02 |
| Software |  |  |
| ImageJ | NIH, Bethesda | https://imagej.nih.gov/ij/ |
| Origin 9.0 | OriginLab | https://www.originlab.com |
| GraphPad Prism 8.0 | GraphPad | https://www.graphpad-prism.cn/ |
| Oligonucleotides | Forward | Reverse |
| Mouse Fabp4 | 5’- AAGGTGAAGAGCATCATAACCCT-3’ | 5’- TCACGCCTTTCATAACACATTCC-3’ |
| Mouse Pparγ | 5’- GGAAGACCACTCGCATTCCTT-3’ | 5’- GTAATCAGCAACCATTGGGTCA-3’ |
| Mouse GAPDH | 5’- AGGTCGGTGTGAACGGATTTG-3’ | 5’- TGTAGACCATGTAGTTGAGGTCA-3’ |
| Mouse Adipoq | 5’- TGTTCCTCTTAATCCTGCCCA-3’ | 5’- CCAACCTGCACAAGTTCCCTT-3’ |
| Mouse Plin1 | 5’- CTGTGTGCAATGCCTATGAGA-3’ | 5’- CTGGAGGGTATTGAAGAGCCG-3’ |
| Mouse Runx2 | 5’- TGGCCGGGAATGATGAGAAC -3’ | 5’- CTGGGATCTCGTCCGCTC -3’ |
| Mouse Alp | 5’- TAACACCAACGCTCAGGTCC-3’ | 5’- TGGATGTGACCTCATTGCCC-3’. |
| Mouse Smad1 | GATCAATCCAGGCTCGGGGA | CGCGCTCTGCTTAGCTCC |
| Mouse Bglap | GCTACCTTGGAGCCTCAGTC | GGCGGTCTTCAAGCCATACT |
|  |  |  |
|  |  |  |
|  |  |  |
|  |  |  |
|  |  |  |
|  |  |  |
|  |  |  |
|  |  |  |

**Fig. S1**


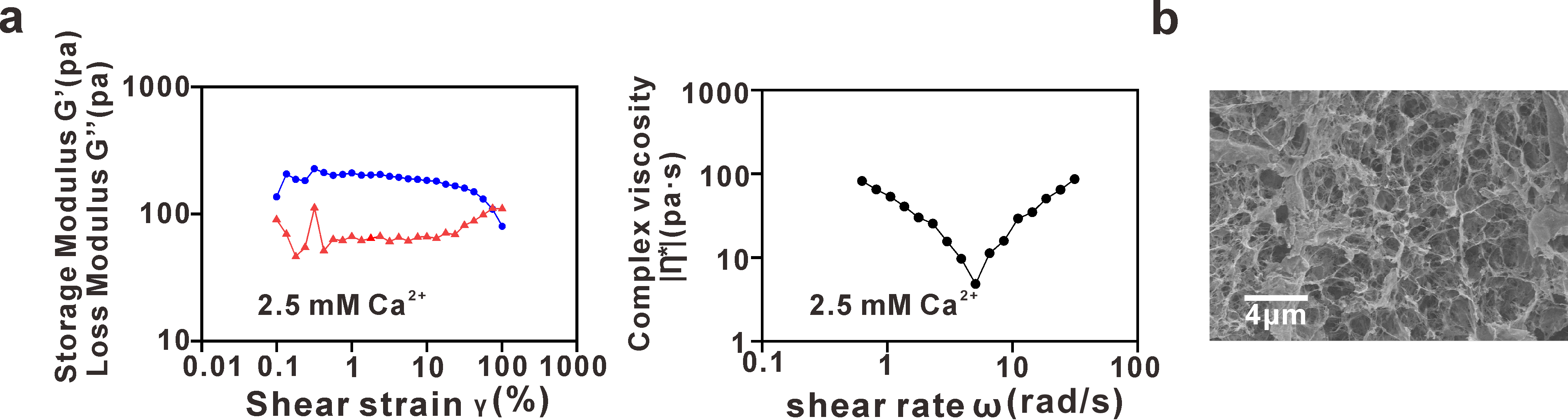


**Fig. S1. a,** Storage and loss modulus as a function of shear strain for soft IPN gel (left); complex viscosity as a function of shear rate for soft IPN gel (right). **b,** Scanning electron micrograph of a IPN hydrogel.

**Fig. S2**


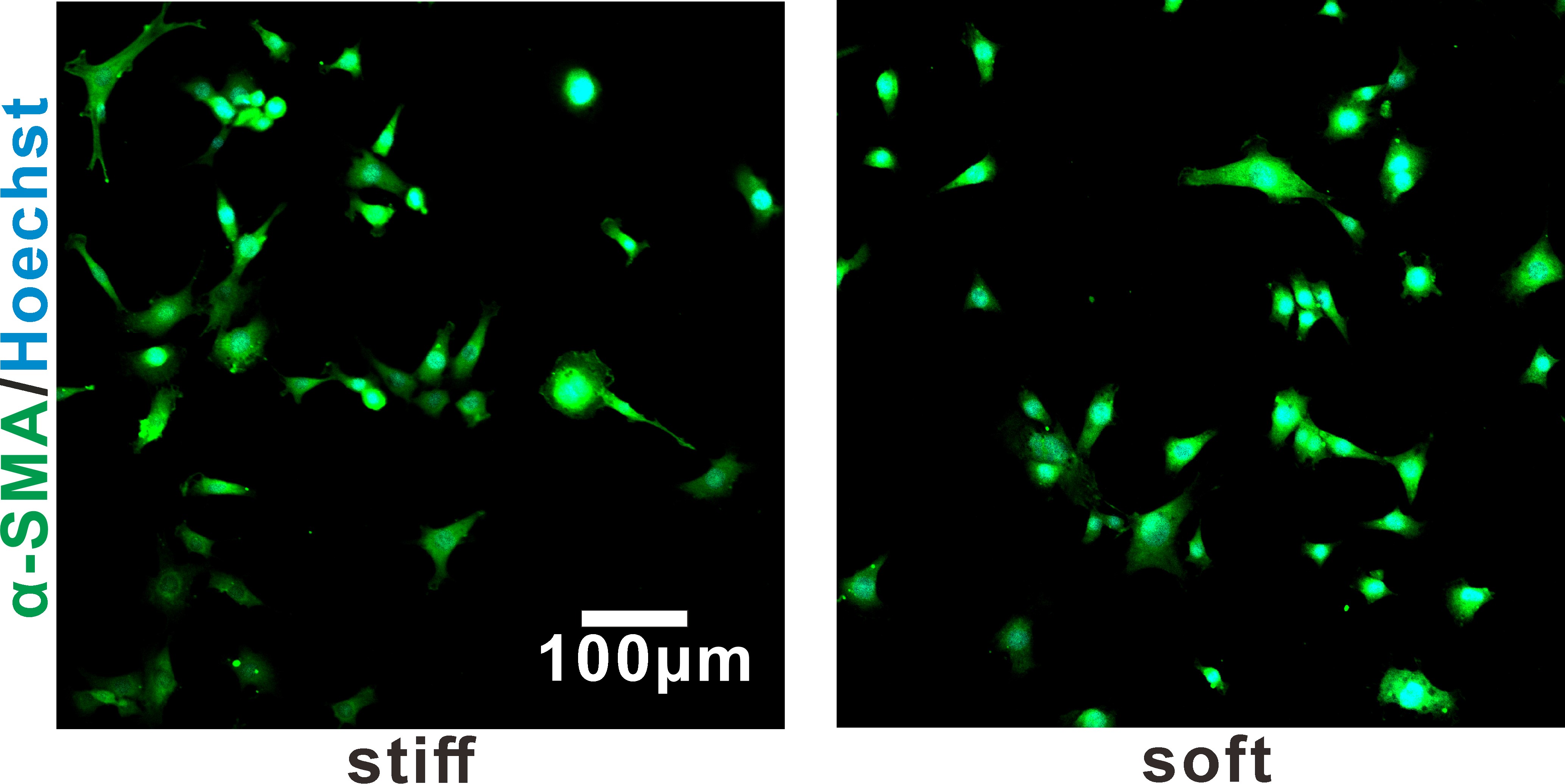


**Fig. S2. Representative images of fibroblasts on the on the stiff and soft PDMS substrates.**

**Fig. S3**


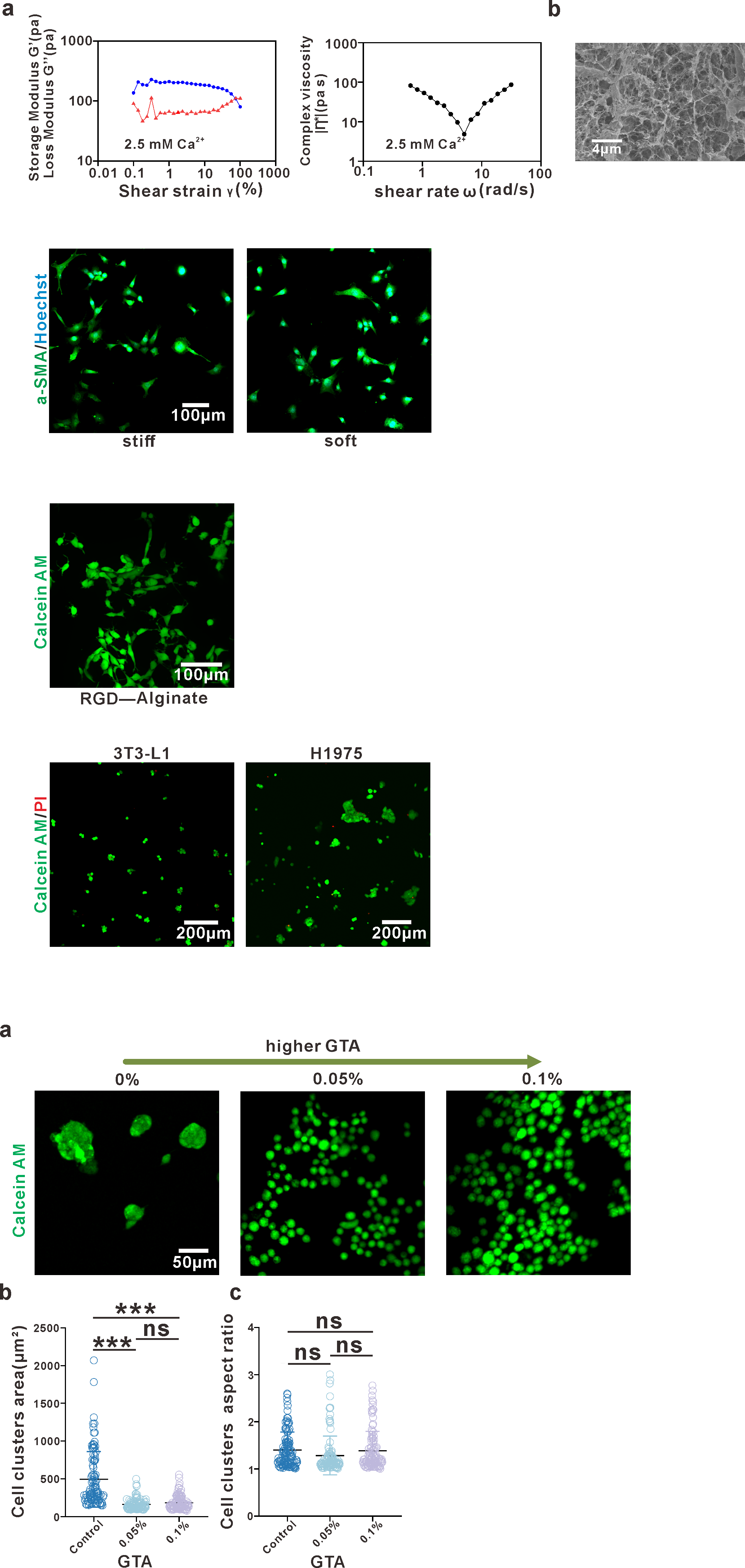


**Fig. S3. Representative images of fibroblasts on the on the 15 mM** **calcium sulfate 824 μM RGD-Alginate substrates.**

**Fig. S4**

**
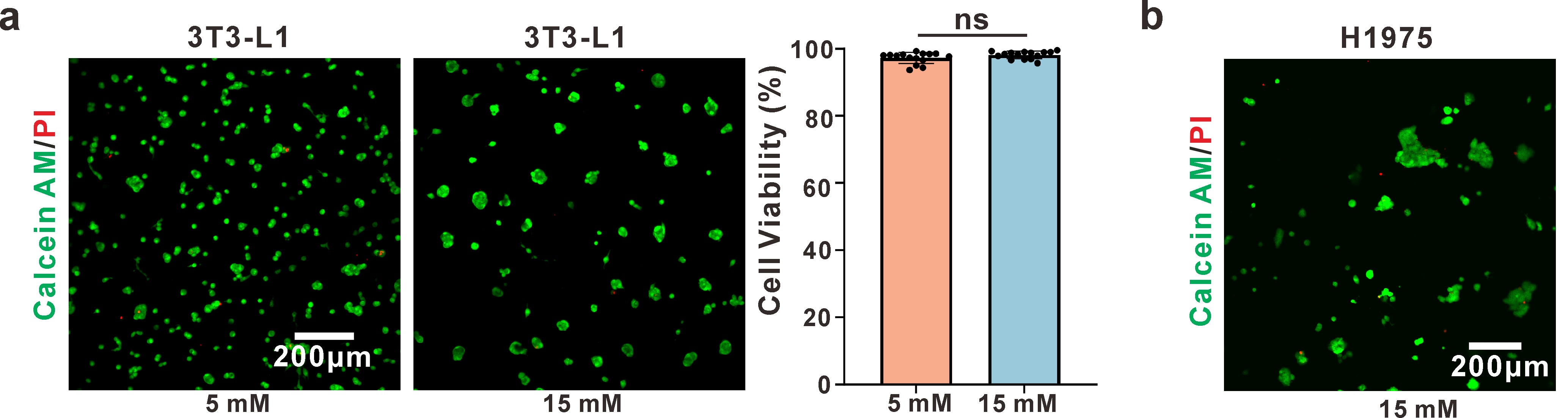
**

**Fig. S4. a,** Live/Dead assay of 3T3-L1 cells cultured on tissue-mimicking hydrogels on day 3 and day 7 with various concentration of calcium. No obvious dead cells were found and there is no significant difference in the cell viability from two groups of cells (calcein-positive area)/ (total stained area) × 100% n=15) **b,** Representative images of live/dead assays performed day 10 of H1975 culture. Data are mean ± SD; *P < 0.05, **P < 0.01, and ***P < 0.001; ns, not significant.

**Fig. S5**

**
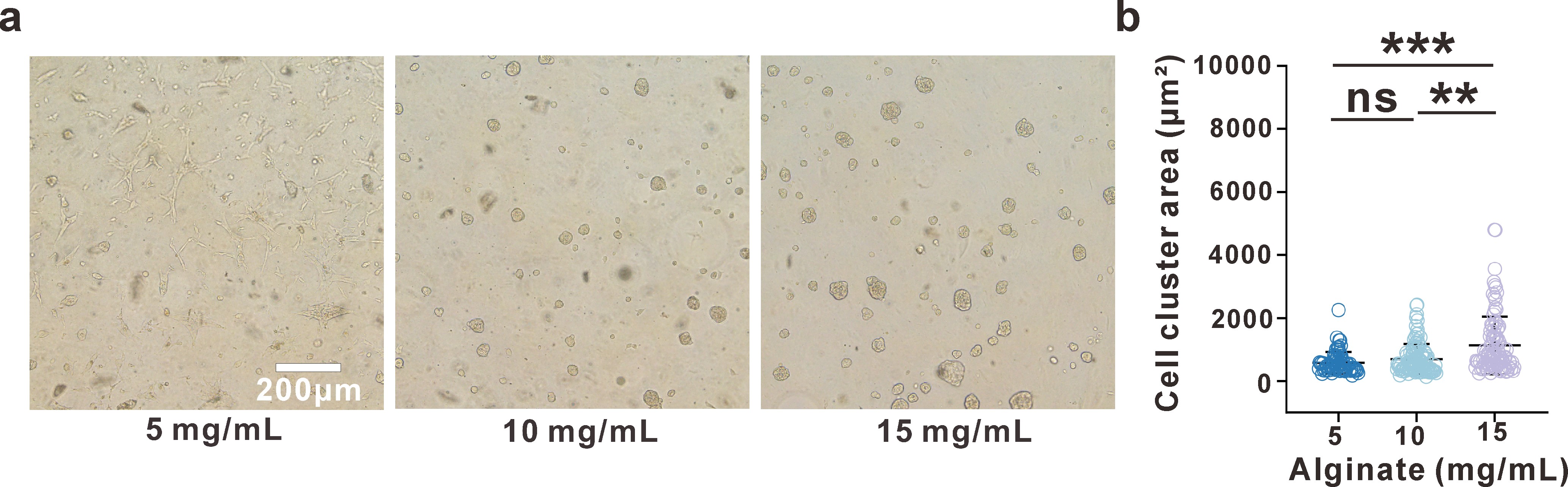
**

**Fig. S5. Cell aggregation on IPN hydrogels with different mesh densities by varying concentrations of alginate. a,** Images of cell aggregates on IPN hydrogels with different alginate concentration on 24 h. **b,** Quantification of cluster areas of cell aggregates on different IPN hydrogels(n≥69). Data are mean ± SD; *P < 0.05, **P < 0.01, and ***P < 0.001; ns, not significant.

**Fig. S6**

**
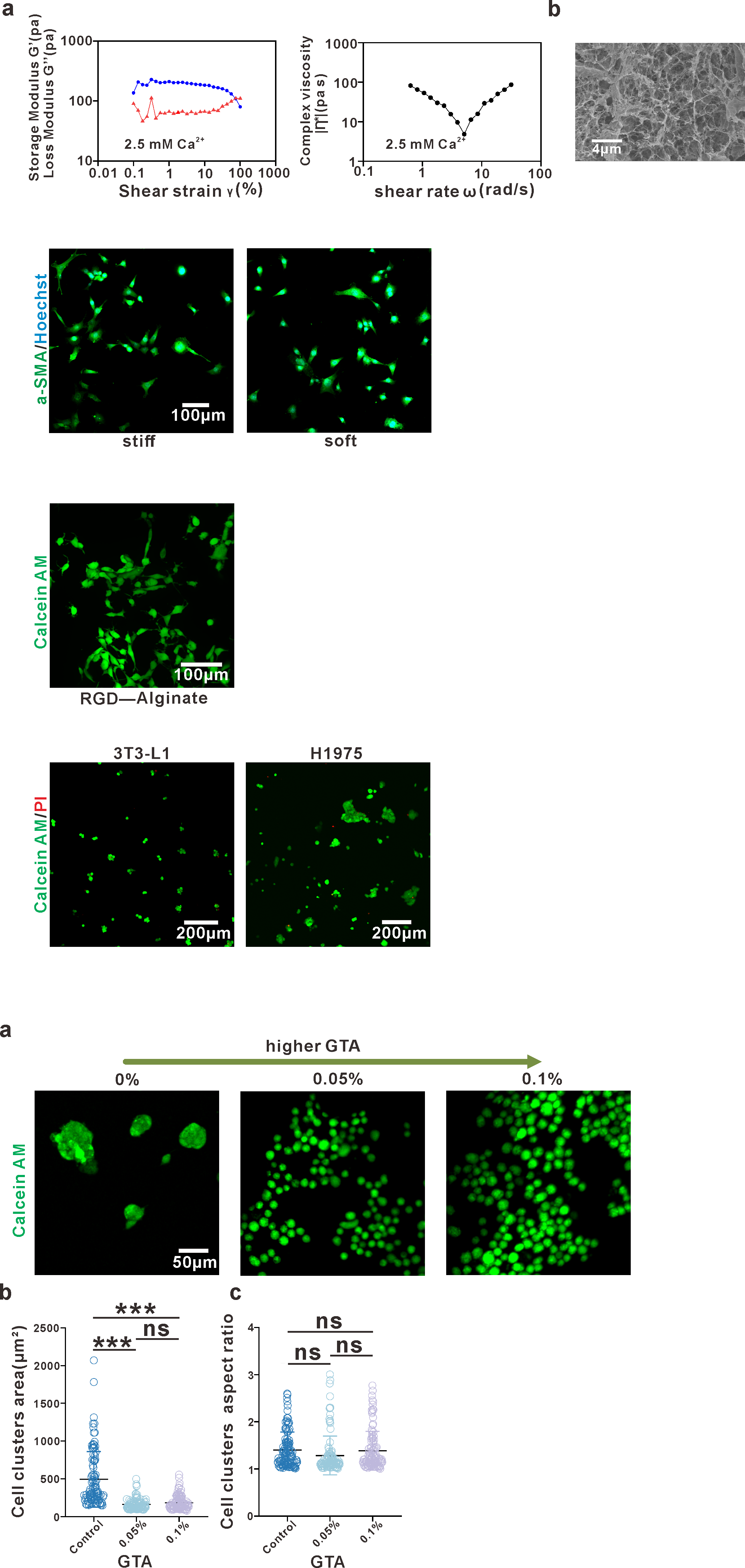
**

**Fig. S6. Representative images of fibroblasts on the glutaraldehyde treated IPN** **hydrogel.** **a,** Inhibition of cell adhesion by 0.05% and 0.1% glutaraldehyde.

**b,** Cell cluster areas(n=101) decreased after treatment of 0.05% and 0.1% glutaraldehyde **c,** Cell cluster aspect ratio(n=111) unchanged after treatment of 0.05% and 0.1% glutaraldehyde. Data are mean ± SD; *P < 0.05, **P < 0.01, and ***P < 0.001; ns, not significant.

**Fig. S7**

**
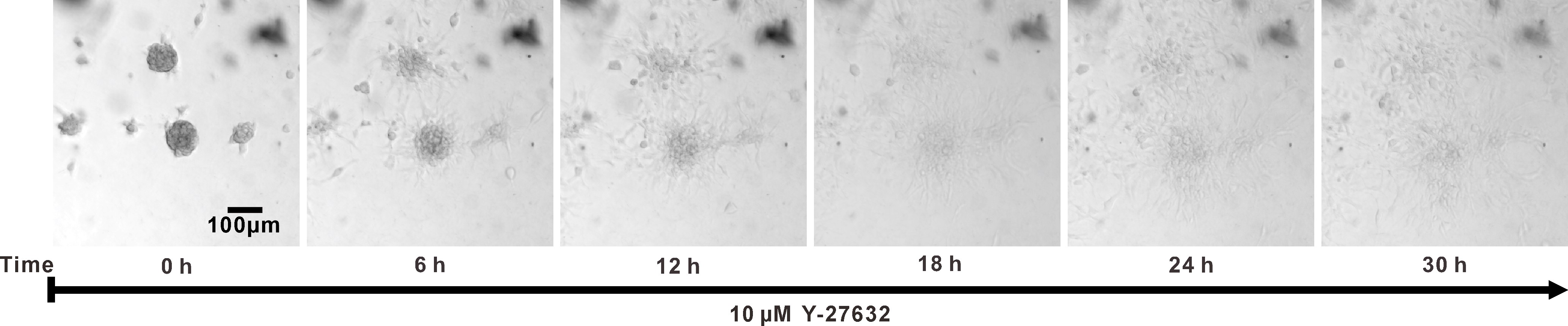
**

**Fig. S7. Time course images showing the dissociation of mesenchymal aggregates upon removal of mechanical cues (by using inhibitor Y-27632). We observed the cells regained the features of spreading and migration.**

**Fig. S8**


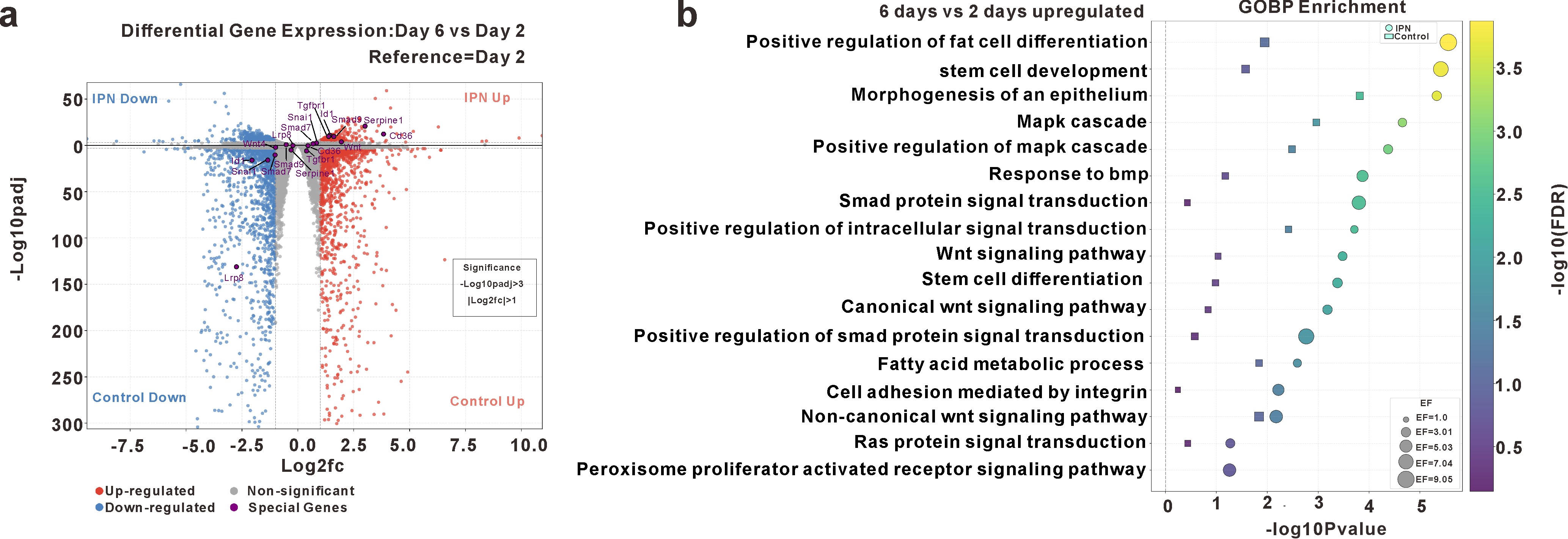


**Fig. S8. a,** The comparative volcano plots demonstrate that while both hydrogel and 2D cultures exhibited temporal gene expression changes over the 4-day period, the hydrogel microenvironment induced an enhanced gene expressions related to WNT pathway, TGF-beta pathway, and Cell cycling. In contrary, the 2D culture condition induced either non-significant changes or inhibit the pathways. **b,** The bubble plots showed the GOBP enrichment of DEGs between 6 days vs 2 Days under either IPN hydrogel condition (circle) or 2D culture condition (square); this showed that IPN hydrogel condition induced more significant changes on gene expression profiles of cultured cells.

**Fig. S9**

**
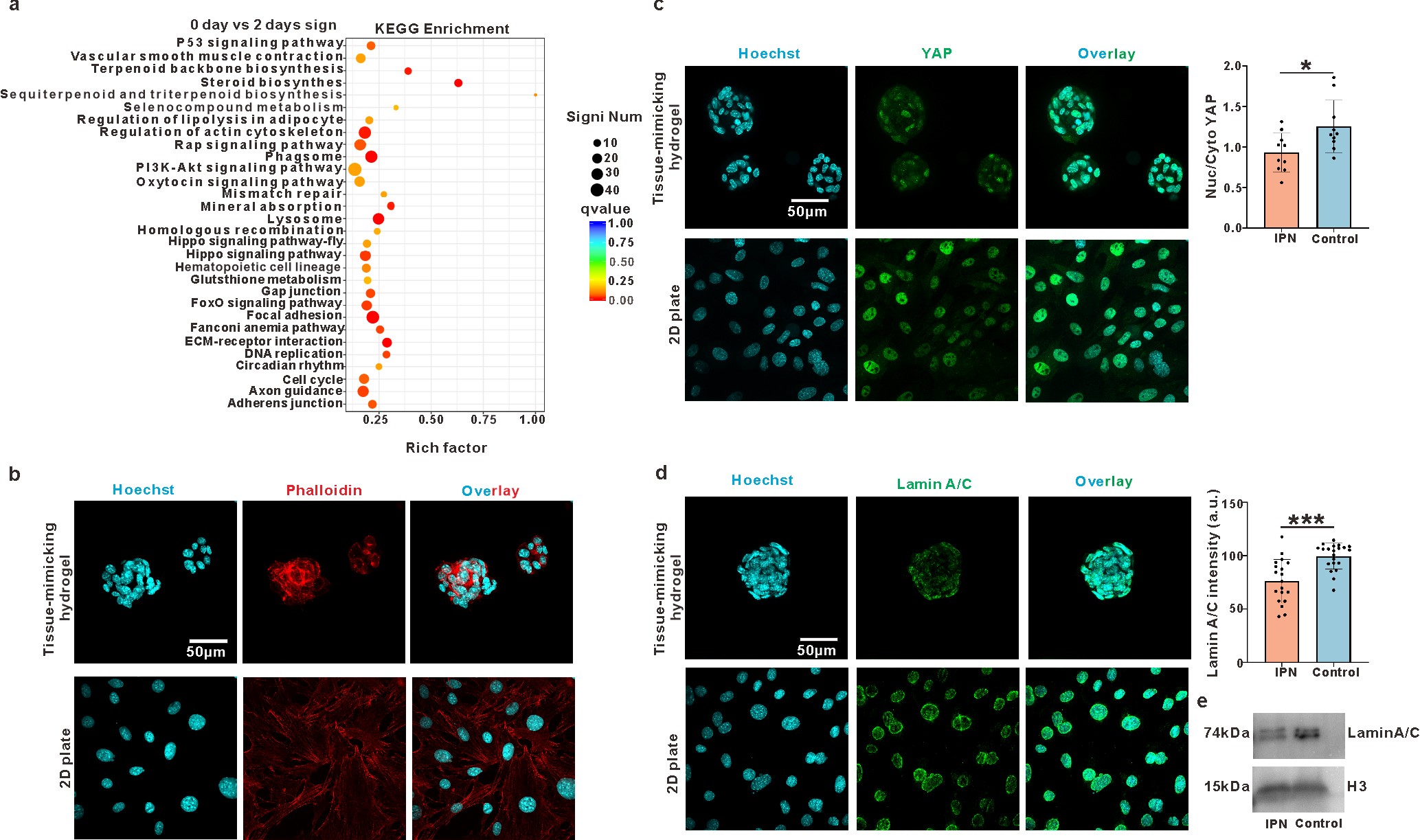
**

**Fig. S9.** **Tissue-Mimicking Hydrogel-Induced Changes in Mechanotransduction Signaling. a,** Bubble plot showing the DEGs enriched Kyoto Encyclopedia of Genes and Genomes (KEGG) pathways terms between cells cultured on Tissue-Mimicking Hydrogel for 0 days and 2 days. **b,** Immunofluorescent staining showing the remodeling of actin cytoskeleton network. **c,** Immunofluorescent staining showing Tissue-Mimicking Hydrogel decreased the nuclear accumulation of YAP(n=10). **d,** Immunofluorescent staining have shown that Tissue-Mimicking Hydrogel decreased the expression of Lamin A/C(n≥19) **e,** Western blot have shown that Tissue-Mimicking Hydrogel decreased the expression of Lamin A/C. Data are mean ± SD; *P < 0.05, **P < 0.01, and ***P < 0.001; ns, not significant.

**Fig. S10**

**
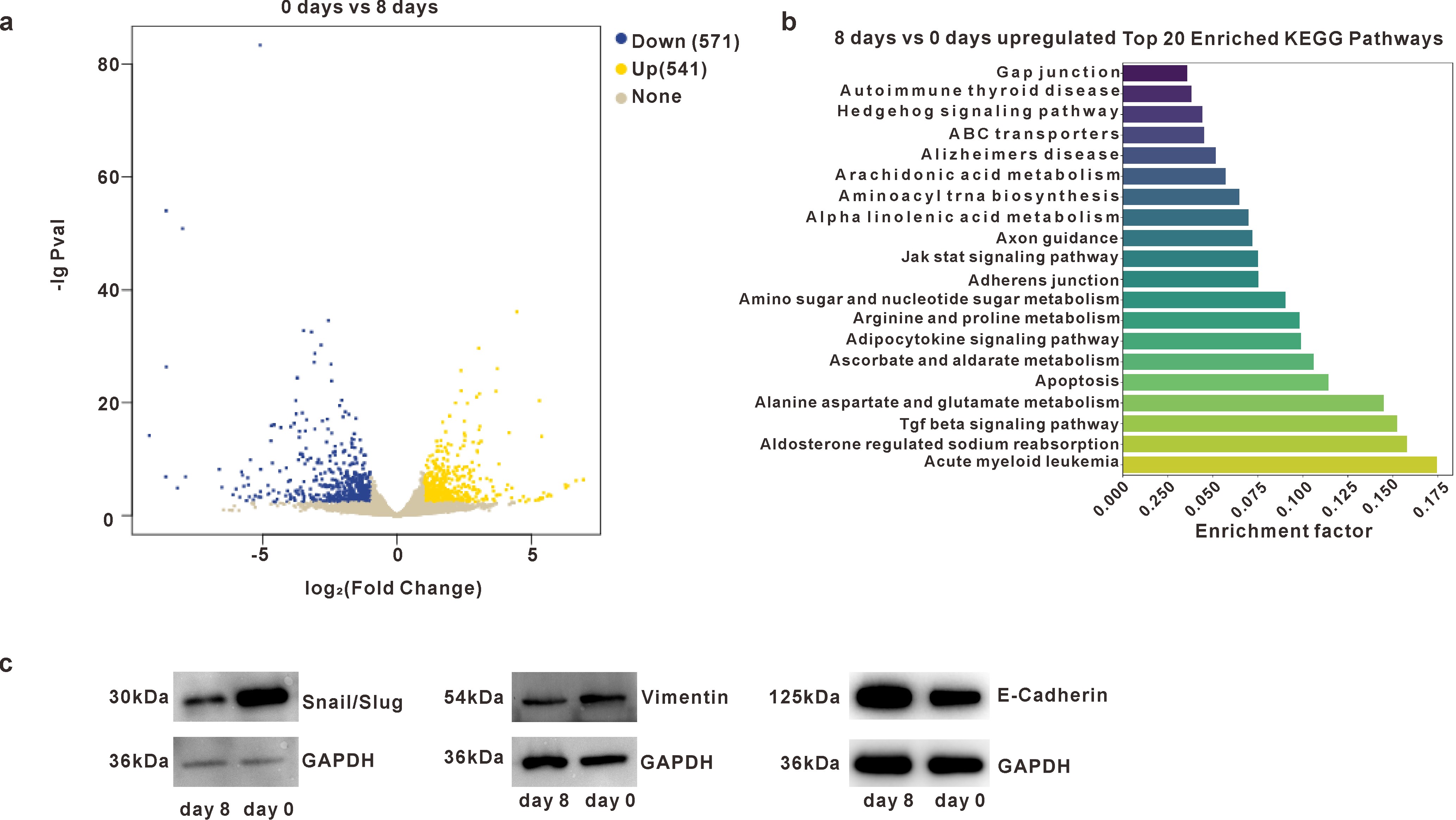
**

**Fig. S10. a,** Volcano plot displaying the significance of changes in gene expression between the cells cultured on Tissue-Mimicking Hydrogel for 8 days and 0 days with adipogenic induction b, Bar plotshowing the DEGs enriched Kyoto Encyclopedia of Genes and Genomes (KEGG) pathways terms between cells cultured on Tissue-Mimicking Hydrogel for 0 days and 8 days**. c,** Western blot have shown that cancer cells on hydrogel expressed genes inhibiting EMT process and genes promoting MET process.

**Movie S1**

**Movie S1.** **Movie showing the formation of mesenchymal aggregates on stiff viscoelastic nonlinear IPN hydrogels within 6-20 h after seeding .**

**Movie S2**

**Movie S2. Movie showing mesenchymal aggregate disaggregation in viscoelastic nonlinear IPN hydrogels after treatment with Y-27632 within 30 h.**
